# Size‐Controlled DNA Tile Self‐Assembly Nanostructures Through Caveolae‐Mediated Endocytosis for Signal‐Amplified Imaging of MicroRNAs in Living Cells

**DOI:** 10.1002/advs.202300614

**Published:** 2023-05-15

**Authors:** Yanan Peng, Zhijun Gao, Bin Qiao, Dongxia Li, Huajie Pang, Xiangde Lai, Qiumei Pu, Rui Zhang, Xuan Zhao, Guangyuan Zhao, Dan Xu, Yuanyuan Wang, Yuxiang Ji, Hua Pei, Qiang Wu

**Affiliations:** ^1^ The Second Affiliated Hospital School of Tropical Medicine Hainan Medical University Haikou 571199 P. R. China; ^2^ Key Laboratory of Emergency and Trauma of Ministry of Education Research Unit of Island Emergency Medicine Chinese Academy of Medical Sciences (No. 2019RU013) Hainan Medical University Haikou 571199 P. R. China; ^3^ Key Laboratory of Tropical Translational Medicine of Ministry of Education School of Pharmacy Hainan Medical University Haikou 571199 P. R. China

**Keywords:** caveolae‐mediated endocytosis, DNA nanostructures, DNA tile self‐assembly technology, living cells, microRNAs, signal‐amplified imaging, size controlled

## Abstract

Signal‐amplified imaging of microRNAs (miRNAs) is a promising strategy at the single‐cell level because liquid biopsy fails to reflect real‐time dynamic miRNA levels. However, the internalization pathways for available conventional vectors predominantly involve endo‐lysosomes, showing nonideal cytoplasmic delivery efficiency. In this study, size‐controlled 9‐tile nanoarrays are designed and constructed by integrating catalytic hairpin assembly (CHA) with DNA tile self‐assembly technology to achieve caveolae‐mediated endocytosis for the amplified imaging of miRNAs in a complex intracellular environment. Compared with classical CHA, the 9‐tile nanoarrays possess high sensitivity and specificity for miRNAs, achieve excellent internalization efficiency by caveolar endocytosis, bypassing lysosomal traps, and exhibit more powerful signal‐amplified imaging of intracellular miRNAs. Because of their excellent safety, physiological stability, and highly efficient cytoplasmic delivery, the 9‐tile nanoarrays can realize real‐time amplified monitoring of miRNAs in various tumor and identical cells of different periods, and imaging effects are consistent with the actual expression levels of miRNAs, ultimately demonstrating their feasibility and capacity. This strategy provides a high‐potential delivery pathway for cell imaging and targeted delivery, simultaneously offering a meaningful reference for the application of DNA tile self‐assembly technology in relevant fundamental research and medical diagnostics.

## Graphical abstract

The size‐controlled 9‐tile nanoarrays can realize direct cytoplasmic delivery via caveolae‐mediated endocytosis to achieve excellent signal‐amplified imaging of intracellular microRNAs, unlike the classical catalytic hairpin assembly (CHA) that enter cells via the clathrin/caveolae‐independent (CCI) pathway and mainstream transport carriers, aiming to solve the problem of enhancing endosomal escape to bypass lysosomal entrapment.

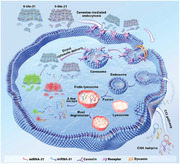

## 1 Introduction

MicroRNAs (miRNAs) are small, noncoding RNAs of ≈22 nucleotides (nt) in length that negatively regulate the translation and degradation of messenger RNA (mRNAs) at the post‐transcriptional level.^[^ ^1^, ^2^ ^]^ The dysregulation of miRNA expression contributes to abnormal proliferation or differentiation of cells, ultimately leading to the occurrence and progression of various diseases.^[^ ^3^ ^]^ In particular, miRNAs are involved in the gene regulation of various pathological pathways in diverse periods of cancer, including growth, invasion, angiogenesis, and immune escape of tumor tissues.^[^ ^4^, ^5^, ^6^, ^7^, ^8^, ^9^, ^10^ ^]^ For this reason, miRNAs have been gradually recognized as a high‐potential tumor biomarker, and related research on miRNA detection in body fluids has been performed more frequently.^[^ ^11^, ^12^, ^13^ ^]^ However, the expression levels of intracellular miRNAs are dynamic and time‐varying;^[^ ^14^, ^15^ ^]^ meanwhile, one type of miRNA is associated with multiple tumors.^[^ ^16^, ^17^, ^18^, ^19^, ^20^ ^]^ Liquid biopsy of miRNAs can reflect total miRNAs in the systemic circulation but fails to pinpoint which tumors are associated with their abnormal expression.^[^ ^13^ ^]^ Therefore, there is an urgent requirement for a technology that can accomplish real‐time, dynamic monitoring of miRNAs at the single‐cell level.

Because of their short sequences, low abundance, easy degradation, and sequence similarity among family members, analytical methods for miRNAs demand high sensitivity and specificity. Traditional detection techniques, including northern blotting, real‐time quantitative polymerase chain reaction (PCR), microarray, capillary gel electrophoresis, and digital PCR, suffer from limited sensitivity, error‐prone amplification, poor reproducibility, or tedious procedures, and are not suitable for miRNA detection in situ at the cellular level.^[^ ^21^ ^]^ Among the newly developed detection technologies, nucleic acid signal amplification techniques can achieve excellent isothermal amplification of miRNAs in living cells.^[^ ^21^, ^22^ ^]^ As a representation of this technology, catalytic hairpin assembly (CHA) possesses the characteristics of a low background signal, simple system composition, and enzyme‐free amplification, rendering it suitable for multiple signal output strategies and contributes to its application at the cellular level.^[^ ^23^, ^24^, ^25^, ^26^, ^27^ ^]^

The pivotal technological difficulty for real‐time dynamic monitoring of intracellular miRNAs has been determining which pathway can efficiently deliver detection systems into the cytoplasm. Nanoparticles enter cells via endocytosis, including phagocytosis, macro‐pinocytosis, as well as clathrin‐mediated, caveolae‐mediated, and clathrin/caveolae‐independent (CCI) endocytosis.^[^ ^28^, ^29^ ^]^ In contrast to the other pathways, caveolae‐mediated endocytosis plays a critical role in the efficient intracellular delivery of nanoparticles with diameters of 60–80 nm. This is due to the frequent bypassing of endo‐lysosomes, in which nanoparticles are coated in caveolin‐stabilized, flask‐shaped plasma membrane invaginations and are directly transported to the cytoplasm.^[^ ^29^, ^30^ ^]^ With their moderate physiological conditions and neutral pH, caveosomes have little impact on the properties and structure of nanoparticles, and the caveolar pathway hardly merges with lysosomes.^[^ ^31^, ^32^, ^33^, ^34^ ^]^ In summary, caveolae‐mediated internalization is an excellent choice for the cellular uptake of nanoparticles.

Consequently, increasing research on intracellular imaging has focused on size optimization of nanoparticles, reflecting that the size is more crucial in nanoparticle internalization among the other physicochemical properties of shape, rigidity, roughness, density, surface charge, surface chemistry, etc.^[^ ^35^, ^36^, ^37^, ^38^, ^39^, ^40^ ^]^ Compared with organic and inorganic materials, DNA nanostructures allow for molecular manipulation at the nanoscale owing to the characteristic of programmability, offering significant superiority in terms of size control.^[^ ^41^, ^42^, ^43^, ^44^ ^]^ Meanwhile, DNA nanostructures are suitable as vectors for intracellular detection systems owing to their simple preparation, optimum biocompatibility, and excellent chemical stability.^[^ ^44^, ^45^, ^46^ ^]^ DNA tile self‐assembly is a crucial branch of DNA nanotechnology and is characterized by the assembly of tiles into arrays. DNA tile self‐assembly nanostructures offer more significant advantages, efficient assembly, and excellent rigidity compared with that of a 3D DNA wireframe‐like DNA tetrahedron, as well as straightforward design and simple preparation without purification compared with that of DNA origami.^[^ ^47^, ^48^, ^49^, ^50^, ^51^ ^]^ In recent years, DNA tile self‐assembly nanostructures have increasingly emerged in relevant research on targeted delivery and therapy, but biosensing has rarely been studied.^[^ ^43^, ^47^, ^52^, ^53^, ^54^ ^]^ Therefore, the core points involve how to build a universal nanoplatform suitable for intracellular imaging combining the inherent merits of DNA tile self‐assembly and nucleic acid signal‐amplified technology.

**Scheme 1:**
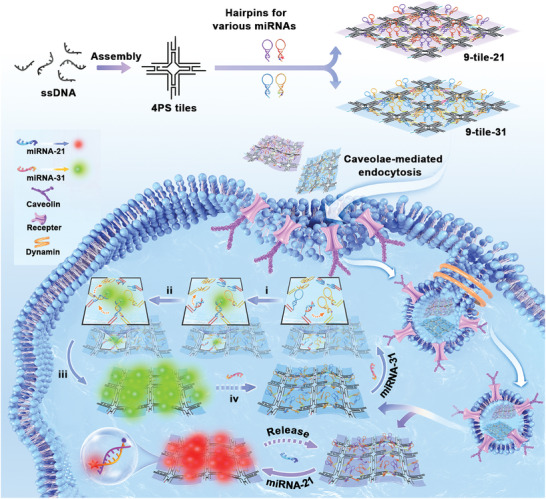
Schematic diagram of caveolae‐mediated internalization of the 9‐tile nanoarrays for amplified imaging of microRNAs in living cells.

In our previous study, using the efficient assembly of DNA tiles, we confined the CHA system to micrometer‐scale DNA tile self‐assembly nanoarrays to achieve a spatially restricted reaction of CHA with accelerated kinetics for the ultrasensitive detection of miRNA‐21 in vitro.^[^ ^55^ ^]^ Herein, based on the above research, we modulated the design principle of the DNA tile self‐assembly and constructed nanoscale, size‐controlled 9‐tile nanoarrays to achieve direct cytoplasmic delivery via caveolae‐mediated endocytosis to realize signal‐amplified imaging of miRNAs in living cells. As shown in **Scheme** **1**, several single DNA strands can self‐assemble into 4‐point‐star (4PS) tiles. Through the introduction of CHA hairpins targeting various miRNAs, nine 4PS tiles were assembled into programmed DNA nanoarrays, named 9‐tile nanoarrays. Each 9‐tile nanoarray, which included 24 pairs of CHA hairpins at specific positions, possessed four nanolattices. Nanoarrays carrying CHA hairpins targeting miRNA‐21 and miRNA‐31 were called 9‐tile‐21 and 9‐tile‐31, respectively. As an example, a structural diagram of 9‐tile‐21 is shown in Figure S1 (Supporting Information), including hairpin loading and specific pairing among the tiles. Several caveolae are expressed in cancer cells, and caveolar endocytosis is size dependent, with sizes ranging from 60 to 80 nm.^[^ ^29^, ^56^ ^]^ Based on this, the 9‐tile nanoarrays can be programmed with a maximum diameter of ≈63 nm to achieve caveolae‐mediated endocytosis. Caveolae‐mediated internalization allows 9‐tile nanoarrays to bypass lysosomal entrapment and achieve direct cytosolic delivery. This facilitates the amplified imaging of miRNAs owing to the increase of 9‐tile nanoarrays in the cytoplasm. As shown in Scheme 1, i) free miRNA‐31 opens hairpin H1 in the nanolattice of 9‐tile‐31, and ii) the double‐stranded complex of hairpins H1 and H2 releases miRNA‐31 into the next pair of CHA hairpins. iii) The CHA reaction is carried out sequentially in the nanolattice. iv) Once the CHA reaction is complete, miRNA‐31 is released into another 9‐tile‐31 to trigger subsequent CHA cascade reactions, which can repeat circularly. The working principle of 9‐tile‐21 is identical to that of 9‐tile‐31. Ultimately, this strategy can achieve dual‐fluorescence imaging of miRNAs at the single‐cell level.

## 2 Results and Discussion

### 2.1 Assembly and Characterization of 9‐Tile Nanoarrays

The 9‐tile nanoarrays were built by assembling tiles 1–9, with each tile containing ten species of DNA strands. The preparation of DNA tiles was a one‐step, straightforward process, only requiring simple heating and natural annealing to room temperature after mixing the DNA strands. To elaborate on the structural composition, each tile was disassembled for verification purposes. As illustrated in Figure S2 (Supporting Information), four N2 strands and one L4 strand built the backbone of tile‐1 from 9‐tile‐21, then four N3 strands were paired with the corresponding N2 strands to generate a 4PS tile with specific cohesive ends. The 3′‐end of hairpin H1 was appended the DNA linker, named L‐H1. L‐H1 assembled onto the 3′‐ or 5′‐end of N2 strands by specific recognition between linkers. Finally, tile‐1 was fabricated and programmed with four site‐specific H1 hairpins. As shown in Figure S3 (Supporting Information), we verified the assembly of tile‐1 by using 6% native polyacrylamide gel electrophoresis (PAGE). Results showed that the backbone of tile‐1 could be assembled by adding four N2 strands individually in the presence of L4. Subsequently, four N3 strands were incorporated into the backbone to construct a 4PS tile without H1. Eventually, the amount of H1 on tile‐1 could be controlled by adding diverse concentrations of L‐H1. Intact tile‐1 carrying four H1 hairpins was assembled by adding 4/13 H1 to the total molar mass, whereas incomplete assembly with less H1. The structural composition and assembly verification of tiles 2–9 are illustrated in Figures S4–S19 (Supporting Information). All oligonucleotide sequences used in this study are listed in Table S1 (Supporting Information).

**Figure 1:**
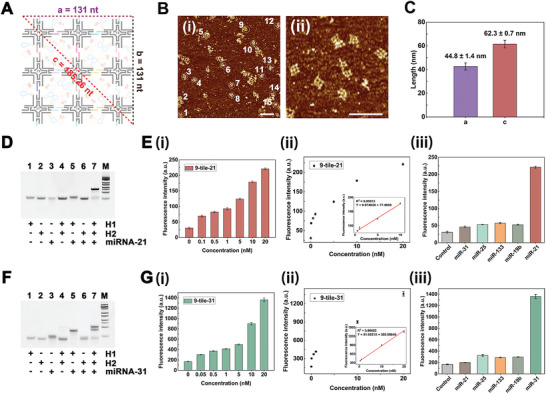
Characterization and microRNA detection using the 9‐tile nanoarrays. A) Scheme of theoretical size calculation for the 9‐tile nanoarrays. Considering the 9‐tile nanoarrays as the square, the side lengths (*a* and *b*) were 131 nucleotides (nt) long. Therefore, the diagonal line *c* was 185.26 nt long according to the right‐triangle formula. B) Atomic force microscopy images at i) lower and ii) higher magnifications of 9‐tile‐21, which were not taken from the same visual field. All scale bars correspond to 200 nm. C) Measurement of 9‐tile‐21 shown in panel (A) was taken using Gwyddion software. The side length *a* was 44.8 ± 1.4 nm and diagonal line *c* is 62.3 ± 0.7 nm. Error bars indicate the standard deviation (*n* = 15). D) 12% native polyacrylamide gel electrophoresis (PAGE) analysis of catalytic hairpin assembly (CHA) reaction progress and relevant reactants for miRNA‐21. E‐i) Fluorescence intensity of 9‐tile‐21 for various concentrations of miRNA‐21; ii) linear analysis of the miRNA‐21 detection results; and iii) method specificity of 9‐tile‐21 was checked using another miRNA family at 2 × 10^−8^ m. F) 12% native PAGE analysis of CHA reaction progress and relevant reactants for miRNA‐31. G‐i) Fluorescence intensity of 9‐tile‐31 for various concentrations of miRNA‐31; ii) linear analysis of the miRNA‐31 detection results; and iii) method specificity of 9‐tile‐31 was checked using another miRNA family at 2 × 10^−8^ m. The DNA marker in panels (D) and (F) was 25–500 bp in size (25, 50, 75, 100, 150, 200, 300, 400, 500 bp). Error bars indicate the standard deviation in panels (E) and (G) (*n* = 3).

Each 4PS tile has four arms, with specific sticky ends at the top, bottom, left, and right sides. As shown in Figures S20 and 21 (Supporting Information), we verified the 9‐tile assembly process through 1% agarose gel electrophoresis (AGE). Results showed that assembly efficiency among the paired tiles was extremely high regardless of whether the tiles were oriented in the horizontal or vertical direction. Through strict identification between the arms of paired tiles, the 9‐tile nanoarrays were successfully constructed with size control. In theory, the side length *a* of the 9‐tile nanoarrays was 131 nt (44.54 nm) long, wherefore the diagonal *c* was calculated to be 185.26 nt (62.99 nm) long using the right‐triangle formula (**Figure** **1**A). Morphology characterization of the 9‐tile nanoarrays, using 9‐tile‐21 as an example, was performed using atomic force microscopy (AFM), as shown in Figure 1B. For the actual measurements using Gwyddion software,^[^ ^57^ ^]^ the lengths of side *a* and diagonal *c* were calculated as 44.8 ± 1.4 and 62.3 ± 0.7 nm, respectively (Figure 1C), which were mostly consistent with the theoretical values. In summary, using the programmability and manipulability of DNA, we designed and fabricated 9‐tile nanoarrays with four‐lattice morphology and a precise size under the preparation conditions of simple heating and annealing.

### 2.2 Fluorescence Detection of MicroRNAs Using 9‐Tile Nanoarrays in vitro

In this study, miRNA‐21 and miRNA‐31 were selected as targets to validate the fluorescence detection capabilities of 9‐tile nanoarrays in vitro. The DNA hairpins, H1 and H2, targeting miRNA‐21 were designed according to the reaction principles of CHA. Results of 12% native PAGE showed that double‐stranded hybridization products of H1 and H2 were generated only in the presence of miRNA‐21 (Figure 1D). In Figure 1E(i), 9‐tile‐21 showed excellent sensitivity to miRNA‐21 at the concentration range of (1–200) × 10^−10^ m, with the lowest detectable concentration (LDC)^[^ ^58^ ^]^ of 1 × 10^−10^ m. Fluorescence detection intensity and the corresponding miRNA‐21 concentrations had a good linear relationship in the regression equation, fitting to *Y* = 9.97483*X* + 77.5055 (*R* ^2^ = 0.99513) (Figure 1E(ii)). Specificity is critical for miRNA identification due to high homology of the miRNA family. We selected interference terms to evaluate the miRNA‐21‐sensing specificity of 9‐tile‐21, including miRNA‐31, miRNA‐25, miRNA‐133, and miRNA‐19b. As shown in Figure 1E(iii), the fluorescence intensity of miRNA‐21 was much higher than that of the interference sequences, whereas all interference sequences exhibited a near‐background fluorescence response.

Similarly, the CHA hairpins, H1 and H2, targeting miRNA‐31 were validated with 12% PAGE (Figure 1F). As shown in Figure 1G(i), 9‐tile‐31 had better detection sensitivity than that of 9‐tile‐21, with an LDC of 5 × 10^−11^ m. The linear fitting equation for the detection intensity and corresponding miRNA‐31 concentration was *Y* = 51.9531*X* + 355.95649 (*R* ^2^ = 0.99603) (Figure 1G(ii)). Although other miRNAs (including miRNA‐21, miRNA‐25, miRNA‐133, and miRNA‐19b) were used as interference, 9‐tile‐31 showed excellent detection specificity (Figure 1G(iii)). These experimental data validated the reliability of 9‐tile nanoarrays for miRNA detection with high sensitivity and specificity and indicated their potency in subsequent intracellular imaging experiments.

### 2.3 Caveolae‐Mediated Internalization of the 9‐Tile Nanoarrays

**Figure 2:**
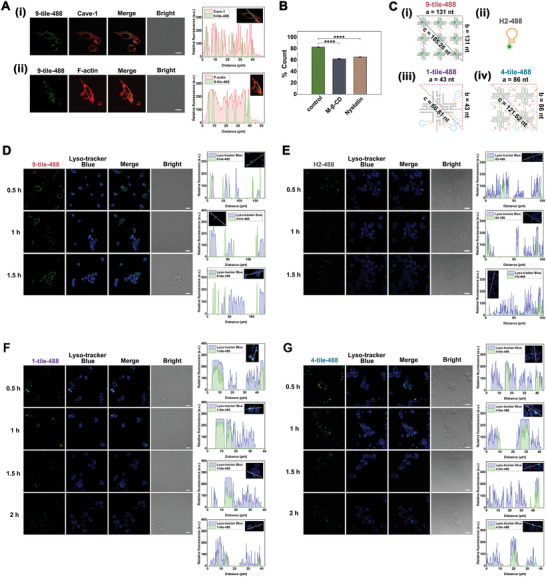
Different pathways of cell uptake for the 9‐tile nanoarrays, DNA hairpins, and two smaller nanoarrays. A) The 9‐tile nanoarrays were labeled with Alexa Fluor 488 and named 9‐tile‐488. HeLa cells were incubated with 9‐tile‐488 (2.5 × 10^−7^ m) for 3 h. Subsequent immunofluorescence staining was performed for Cave‐1 (Texas red) or F‐actin (Texas red). Confocal laser scanning microscopy (CLSM) imaging of HeLa cells for Alexa Fluor 488 and Texas Red. Yellow spots indicate the co‐localization of 9‐tile‐488 with i) Cave‐1 or ii) F‐actin. Analysis of the fluorescence co‐localization of 9‐tile‐488 with i) Cave‐1 or ii) F‐actin in the inset figures using ImageJ software. All scale bars correspond to 20 µm. B) Analysis of the cellular uptake for 9‐tile‐488 after pretreatment with the caveolae inhibitors, nystatin (1 × 10^−5^ m) or methyl‐*β*‐cyclodextrin (M‐*β*‐CD) (5 × 10^−3^ m), for 1 h at 37 °C. Fluorescence intensity was determined via flow cytometry. ****p < 0.0001 (two‐sample *t*‐test) (*n* = 3). Error bars indicate the standard deviation (*n* = 3). C) Schemes of theoretical size calculations for i) 9‐tile‐488, iii) 1‐tile‐488, and iv) 4‐tile‐488. ii) Structural diagram of H2‐488. The hairpin H2 and tile‐1 were labeled with Alexa Fluor 488 and named H2‐488 and 1‐tile‐488, respectively. The 4‐tile‐488, labeled with Alexa Fluor 488, was composed of tile‐1, tile‐2, tile‐4, and tile‐5. From the results of Figure 1A, the diagonal line *c* of 9‐tile‐488 was 185.26 nt long. Considering 1‐tile‐488 and 4‐tile‐488 as the squares, the diagonal lines *c* were 60.81 nt (20.68 nm) and 121.62 nt (41.35 nm) long, respectively, according to the right‐triangle formula. CLSM imaging and analysis of the fluorescence co‐localization between Lyso‐tracker Blue and D) 9‐tile‐488 (2.5 × 10^−7^ m), E) H2‐488 (2.5 × 10^−7^ m), F) 1‐tile‐488 (2.5 × 10^−7^ m), and G) 4‐tile‐488 (2.5 × 10^−7^ m) for 0.5, 1, and 1.5 h in HeLa cells at 37 °C. All scale bars correspond to 20 µm.

Except for caveolar endocytosis, most internalization pathways confine nanoparticles to endosomes, where they merge with lysosomes to generate endo‐lysosomes that possess numerous digestive enzymes and an acidic environment.^[^ ^59^ ^]^ The degradation of nanoparticles in endo‐lysosomes is high, whereas their escape efficiency into the cytoplasm is extremely low.^[^ ^60^ ^]^ Therefore, direct cytosol delivery strategies, such as caveolar endocytosis, which avoid entrapment by lysosomes, have high potential for research at the cellular level. Therefore, we limited the maximum diameter of the 9‐tile nanoarrays to ≈63 nm to achieve caveolae‐mediated internalization. To validate the endocytosis pathway, we analyzed the fluorescence co‐localization of 9‐tile nanoarrays with caveolin‐1 (Cave‐1) or the actin cytoskeleton, which are proteins involved in caveolar trafficking.^[^ ^61^, ^62^ ^]^ The 9‐tile nanoarrays labeled with Alexa Fluor 488 were named 9‐tile‐488. After adding 9‐tile‐488 (green) to HeLa cells (human cervical cancer cells) for 3 h, Cave‐1 (red) and F‐actin (red) were marked via cellular immunofluorescence (**Figure** **2**A). Results showed that there was a wide range of fluorescence co‐localization of 9‐tile‐488 with Cave‐1 (Figure 2A(i)) and 9‐tile‐488 with F‐actin (Figure 2A(ii)).

Caveolae‐mediated endocytosis depends on lipid rafts and is subjected to endocytic inhibitors, such as filipin, genistein, nystatin, and methyl‐*β*‐cyclodextrin (M‐*β*‐CD).^[^ ^63^ ^]^ In this study, we chose nystatin and M‐*β*‐CD to pretreat HeLa cells for 1 h. Flow cytometry analysis showed a significant decrease in the internalization percentage of 9‐tile‐488 after inhibitor treatment (Figure 2B). This indicated that 9‐tile‐488 entered cells via the caveolae‐mediated internalization pathway.

We analyzed the fluorescence co‐localization of 9‐tile‐488 and lysosomes to certify that 9‐tile‐488 bypassed lysosomal entrapment. In Figure 2D, results showed that there was little co‐localization of 9‐tile‐488 with the lysosomal dye, Lyso‐tracker Blue, during the co‐incubation periods of 0.5, 1, and 1.5 h. To confirm that the size was the crucial parameter for caveolar endocytosis, we constructed other DNA tile self‐assembly nanoarrays with diverse sizes (Figure 2C), including 1‐tile (maximum diameter = 20.68 nm) and 4‐tile nanoarrays (maximum diameter = 41.35 nm). These were used to observe the performance of intracellular trafficking in lysosomes. Results showed almost absolute co‐localization between 1‐tile‐488 (green) and Lyso‐tracker Blue (blue) at 0.5 and 1 h, only a small number of 1‐tile‐488 escaped from the endo‐lysosome at 1.5 h, and the fluorescence value decreased substantially at 2 h (Figure 2F). The fluorescence co‐localization of 4‐tile‐488 was similar to that of 1‐tile‐488 (Figure 2G). The above results illustrated that smaller 1‐tile and 4‐tile nanoarrays did not satisfy caveolar internalization compared with that of 9‐tile nanoarrays.

To further illustrate necessity for the construction of 9‐tile nanoarrays, the internalization mechanism of classical CHA hairpins was verified. This mechanism was defined as CCI pathways in previous research and found to be involved in fusion with lysosomes.^[^ ^29^ ^]^ We chose CHA hairpin H2 modified with Alexa Fluor 488 to observe fluorescence co‐localization with lysosomes during co‐incubation periods of 0.5, 1, and 1.5 h. Results showed that only a small number of H2‐488 appeared beyond the range of lysosomal labeling until 1.5 h, and green fluorescence diminished substantially over time (Figure 2E). This indicated that most of the H2‐488 was trapped and degraded by the endo‐lysosome and that only a few escaped into the cytoplasm. Based on the above results, we concluded that caveolae‐mediated internalization could be achieved by controlling the size of 9‐tile nanoarrays. This direct cytoplasmic transport could avoid the endo‐lysosome pathway and increase the effective concentration of 9‐tile nanoarrays in the cytoplasm.

### 2.4 Optimization of Imaging Parameters for 9‐Tile Nanoarrays in Cancer Cells

**Figure 3:**
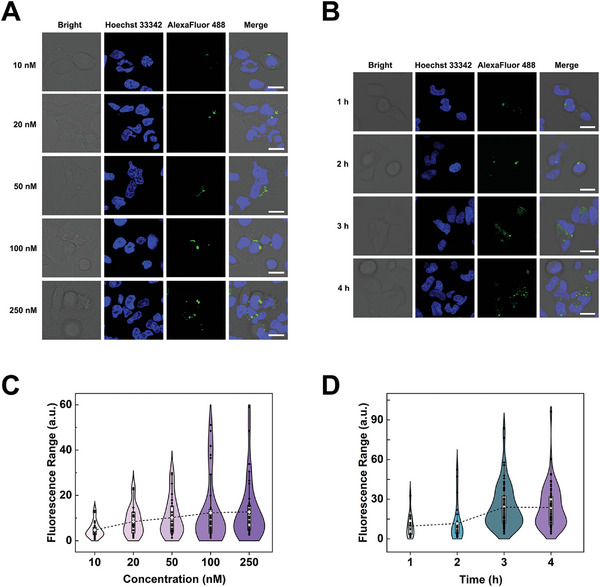
Condition optimization for 9‐tile nanoarray imaging in living cells. Confocal laser scanning microscopy (CLSM) imaging of HeLa cells treated with 9‐tile‐31 at A) different concentrations and B) 1 h intervals. All scale bars correspond to 20 µm. Statistical violin plot analysis of the fluorescence signal distribution of CLSM images (*n* = 3) of 9‐tile‐31 for each C) treatment concentration and D) incubation time. Dotted lines indicate the mean values connected for each group.

To prime imaging performance, imaging parameters for 9‐tile nanoarrays in cancer cells were optimized. First, 9‐tile‐31 was used to evaluate the ideal working concentration for HeLa cells. HeLa cells were treated with five concentrations of 9‐tile‐31 (0.1 × 10^−7^, 0.2 × 10^−7^, 0.5 × 10^−7^, 1 × 10^−7^, and 2.5 × 10^−7^ m) for 4 h, after which the nuclei were labeled with Hoechst 33342. Results from confocal laser scanning microscopy (CLSM) imaging revealed that when the 9‐tile‐31 concentration increased, green fluorescence steadily increased, indicating that the effect of miRNA‐31 imaging (Alexa Fluor 488) increased (**Figure** **3**A). To acquire more accurate data, we chose three CLSM images of each concentration group stochastically and analyzed the fluorescence signal distribution using ImageJ software.^[^ ^64^ ^]^ The analysis results are displayed as a violin statistics chart. The quantity and intensity of the fluorescence signals reached a plateau at 1 × 10^−7^ m, showing that this concentration was the optimal working concentration for 9‐tile‐31 (Figure 3C).

In addition, the 9‐tile nanoarrays required several attempts to enter the cells and proceed with the amplified imaging of miRNAs. To determine the optimal reaction time, we observed the imaging effects of 9‐tile‐31 on HeLa cells at four time points (1, 2, 3, and 4 h). CLSM imaging results showed that the amount and intensity of green fluorescence in HeLa cells increased with time, indicating that the miRNA‐31 imaging (Alexa Fluor 488) effect of 9‐tile‐31 improved over time (Figure 3B). We analyzed three random CLSM images using ImageJ software and found that the fluorescence intensity reached a plateau at 3 h (Figure 3D). Owing to the importance of reaction time for intracellular imaging, 9‐tile‐21 for miRNA‐21 imaging in MCF‐7 cells (human breast cancer cells) was performed at different times, and the optimal reaction time found to be 3 h (Figure S22, Supporting Information). In summary, the optimal working concentration of 9‐tile nanoarrays for intracellular imaging of tumor cells was 1 × 10^−7^ m and optimal reaction time was 3 h. Our strategy has certain advantages when compared with that of previous research on miRNA detection, such as having a shorter optimal imaging time and requiring lower working concentrations. A detailed comparison is presented in Table S2 (Supporting Information). These two parameters were used in subsequent intracellular imaging experiments.

### 2.5 Signal‐Amplified Imaging of MicroRNAs Using 9‐Tile Nanoarrays

Additional attention must be paid to the stability of nanoparticles in physiological environments when research is conducted at the cellular level. The thermodynamic stability of 9‐tile nanoarrays is crucial for living cells. Because the shortest sequences of 9‐tile nanoarrays are the sticky ends of paired tiles, it is vital to control the thermodynamic parameters of two‐state melting hybridization. Therefore, the melting temperature of the paired tiles was strictly kept within the range of 36.8‐50.6 °C based on specific recognition to ensure excellent thermodynamic stability in the intracellular environment of 37 °C (Table S3, Supporting Information). In addition, serum stability investigations can provide an accurate assessment of physiological stability. After adding 10% fetal bovine serum (FBS) to the 9‐tile nanoarrays or hairpin H1 for the various incubation periods at 37 °C, results showed that significant degradation was observed for H1 at 6 h, whereas the serum stability of 9‐tile nanoarrays was excellent up to 8 h (Figure S23, Supporting Information). This indicated that the CHA system could be preserved to avoid degradation after fixing onto the DNA nanoarrays, ultimately boosting the working concentration of the CHA system in cells.

**Figure 4:**
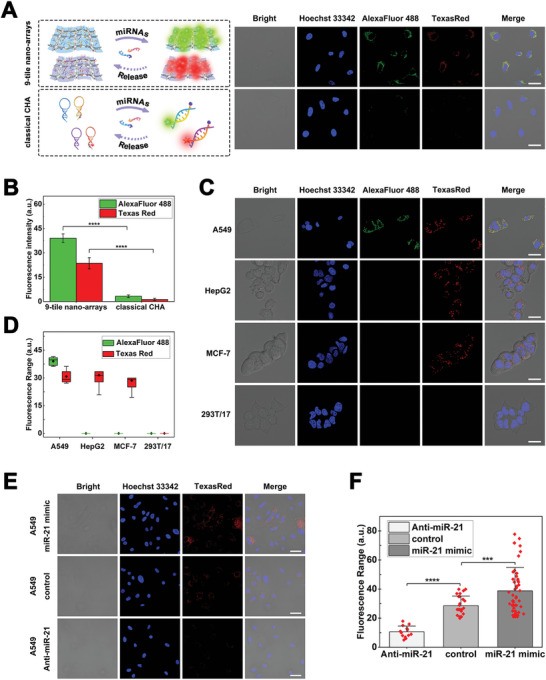
The 9‐tile nanoarrays used for intracellular imaging of microRNAs and comparisons with the classical catalytic hairpin assembly. A) Confocal laser scanning microscopy (CLSM) imaging of HeLa cells treated with 9‐tile nanoarrays and classical catalytic hairpin assembly (CHA). The 9‐tile nanoarrays included 9‐tile‐31(Alexa Fluor 488) and 9‐tile‐21(Texas Red), and classical CHA included CHA‐31 (Alexa Fluor 488) and CHA‐21 (Texas Red), which contained hairpins targeting miRNA‐31 and miRNA‐21, respectively. All scale bars correspond to 20 µm. B) Corresponding statistical histogram analysis of the fluorescence intensity from five random points in panel (A) using ImageJ software. Error bars indicate the standard deviation (*n* = 5). ****p < 0.0001 (two‐sample *t*‐test, 9‐tile nanoarrays vs classical CHA). C) CLSM imaging of miRNAs for 9‐tile‐31 (1 × 10^−7^ m) and 9‐tile‐21 (1 × 10^−7^ m) in different cell lines. All scale bars correspond to 20 µm. D) Corresponding statistical boxplot analysis of the fluorescence range from five random points in panel (C) using ImageJ software. Black rhombic points were the mean values of fluorescence intensity. Error bars indicate the standard deviation (*n* = 5). E) CLSM imaging of intracellular miRNA‐21 by 9‐tile‐21 in miRNA‐21 mimic‐pretreated, unpretreated (control), and anti‐miRNA‐21‐pretreated A549 cells. All scale bars correspond to 20 µm. F) Corresponding statistical histogram analysis of the fluorescence signal distribution of CLSM images in panel (E) using ImageJ software. ****p < 0.0001 (two‐sample *t*‐test, control vs anti‐miRNA‐21). ***p < 0.001 (two‐sample *t*‐test, control vs miRNA‐21 mimic). Error bars indicate the standard deviation (*n* values were 13, 25, and 49 for anti‐miR‐21, control, and miR‐21 mimic, respectively).

Compared with classical CHA, 9‐tile nanoarrays can protect hairpins from degradation while achieving a high working concentration in the cytoplasm due to caveolae‐mediated internalization, ultimately improving CHA reaction effects. In a comparative experiment using CLSM imaging, we found that the imaging effect of miRNAs on 9‐tile nanoarrays in HeLa cells was significantly better than that of classical CHA (**Figure** **4**A). Through measuring the fluorescence intensity of five random points, the mean fluorescence intensity of 9‐tile nanoarray imaging for miRNA‐31 (Alexa Fluor 488) and miRNA‐21 (Texas Red) were 39.0 ± 2.7 and 23.6 ± 3.5, respectively, whereas those of classical CHA were 3.3 ± 0.8 and 1.3 ± 0.6, respectively (Figure 4B). Using the statistical method of the two‐sample *t*‐test to compare fluorescence intensity, the values of 9‐tile nanoarrays were significantly higher than those of classical CHA (*p* < 0.0001, *n* = 5). Thus, we conclude that our strategy could achieve more stable and preferable signal‐amplified imaging of intracellular miRNAs than that of classical CHA.

We compared the imaging effects in different tumor cell lines to further confirm the feasibility of our strategy. Compared with normal cells, miRNA‐31 and miRNA‐21 expressions were upregulated in both A549^[^ ^65^, ^66^ ^]^ (human lung cancer cells) and HeLa cells,^[^ ^67^, ^68^ ^]^ whereas miRNA‐21 expression was upregulated but miRNA‐31 downregulated in HepG2^[^ ^69^, ^70^ ^]^ (human liver cancer cells) and MCF‐7 cells^[^ ^71^ ^]^ (human breast cancer cells), with 293T/17 cells^[^ ^68^ ^]^ (human renal epithelial cells) as the control. In previous experiments, miRNA‐31 (Alexa Fluor 488) and miRNA‐21 (Texas Red) were detected simultaneously in HeLa cells using 9‐tile‐31 and 9‐tile‐21, respectively. As shown in Figure 4C, the fluorescent signals of miRNA‐31 and miRNA‐21 were both detected in A549 cells, and only miRNA‐21 detected in HepG2 and MCF‐7 cells, whereas 293T/17 cells did not show any fluorescent signal under identical imaging parameters. By selecting five random points on the CLSM images for the analysis of fluorescence intensity, the statistical boxplot showed diverse fluorescence ranges corresponding to the expression levels of miRNAs in different tumor cell lines that were consistent with previous research (Figure 4D).

The expression levels of intracellular miRNAs are often dynamic and shifty during tumorigenesis and progression, and real‐time analysis and monitoring of miRNAs using intracellular imaging technology are meaningful for the early diagnosis and therapeutic evaluation of tumors. Thus, the real‐time response of 9‐tile nanoarrays to up‐ or downregulated expression levels of miRNA‐21 was observed and evaluated. As shown in Figure 4E, compared with control cells, more prominent fluorescence spots were observed in miRNA‐21 mimic‐pretreated A549 cells, but even less in anti‐miRNA‐21‐pretreated A549 cells. As shown in Figure 4F, the statistical histogram of fluorescence signal distribution in three groups showed a gradual upward trend with an increase in the expression level of miRNA‐21. The above results illustrate the feasibility and potential of this strategy for real‐time, dynamic monitoring of miRNAs in different tumor or identical cells at different periods.

### 2.6 Cytotoxicity Evaluation of 9‐Tile Nanoarrays

**Figure 5:**
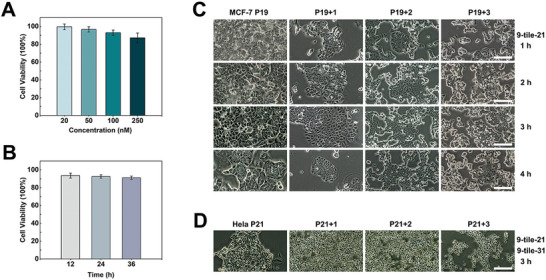
Cytotoxicity evaluation of the 9‐tile nanoarrays. A) Cell viability after incubation with 9‐tile nanoarrays in diverse concentration gradients in HeLa cells for 4 h at 37 °C. Error bars indicate the standard deviation (*n* = 3). B) Comparison of cell viability among the different incubation times (12, 24, and 36 h) in HeLa cells. The dose of 9‐tile nanoarrays was 1 × 10^−7^ m. Error bars indicate the standard deviation (*n* = 3). C) MCF‐7 cells were incubated with 9‐tile‐21 (1 × 10^−7^ m) for 1, 2, 3, and 4 h. After intracellular imaging of miRNA‐21 for 9‐tile‐21, the cells were passaged for three generations and observed via light microscopy (LM). All scale bars correspond to 400 µm. D) HeLa cells were incubated with 9‐tile‐21 (1 × 10^−7^ m) and 9‐tile‐31 (1 × 10^−7^ m) for 3 h. After dual‐fluorescence imaging of miRNA‐21 and miRNA‐31 for 9‐tile‐21 and 9‐tile‐31, the cells were passaged for three generations and observed via LM. All scale bars correspond to 400 µm.

DNA nanostructures exhibit excellent biocompatibility and low cytotoxicity. This research chose HeLa cells to evaluate the cytotoxicity of 9‐tile nanoarrays with the CCK‐8 test. First, we evaluated cell viability for different concentrations of 9‐tile nanoarrays. Results showed that the cell viability values were 99.6%, 96.7%, 93.0%, and 87.2%, respectively, after HeLa cells were incubated with four concentration gradients of 9‐tile nanoarrays (0.2 × 10^−7^, 0.5 × 10^−7^, 1 × 10^−7^, and 2.5 × 10^−7^ m) for 4 h (**Figure** **5**A). We then evaluated cell viability at different incubation times. Because the concentration of 9‐tile nanoarrays was 1 × 10^−7^ m in the intracellular imaging experiments, we selected this concentration for cell viability evaluation at 12, 24, and 36 h. As shown in Figure 5B, there was a small influence on cell viability when the incubation time of 9‐tile nanoarrays was extended, and results of the three groups were 93.6%, 92.7%, and 91.1%, respectively. Thus, we conclude that 1 × 10^−7^ m 9‐tile nanoarrays was mostly nontoxic to HeLa cells within 36 h.

MCF‐7 and HeLa cells were used to evaluate whether 9‐tile nanoarrays displayed side effects on tumor cells. MCF‐7 cells were incubated with 9‐tile‐21 for 1, 2, 3, and 4 h, followed by miRNA‐21 imaging. The cells exhibited satisfactory proliferation ability and cell state after being passaged for three generations, and the substrate was clean without abnormal cell secretion as observed via light microscopy (LM) (Figure 5C). In addition, we evaluated cell viability after dual‐fluorescence imaging of the miRNAs. The 9‐tile‐21 and 9‐tile‐31 were added to HeLa cells and incubated for 3 h. After simultaneous imaging of miRNA‐21 and miRNA‐31, the cells were passaged for three generations. As shown in Figure 5D, there was a little effect on the growth and proliferation of HeLa cells. These results indicated that 9‐tile nanoarrays have extremely low cytotoxicity, good safety, and high potential for miRNA imaging in living cells.

## 3 Conclusion

In this study, we devised and developed size‐controlled 9‐tile nanoarrays that could satisfy caveolae‐mediated internalization for efficient cytoplasmic transport, ultimately achieving optimal signal‐amplified imaging of miRNAs at the single‐cell level. Results of AFM characterization confirmed that the morphology and size of 9‐tile nanoarrays were consistent with the theoretical design, and fluorescence detection of miRNAs in vitro confirmed their sensitivity and specificity. Cellular uptake experiments indicated that caveolae‐mediated internalization could be achieved by controlling the size of 9‐tile nanoarrays. Under the optimal imaging conditions of miRNAs, the 9‐tile nanoarrays exhibited significant advantages in the imaging of miRNAs compared with classical CHA because of their preferable physiological stability and higher delivery efficiency. The miRNA imaging effects of 9‐tile nanoarrays in different cell lines and identical cells at different periods were consistent with the actual expression levels of miRNAs, which adequately demonstrated the feasibility of dynamic monitoring. The cytotoxicity of 9‐tile nanoarrays was exceedingly low, as confirmed using CCK‐8 tests and continuous cell culture after miRNA imaging. In conclusion, because of the programmability and maneuverability of the DNA tile self‐assembly technology on the nanoscale, the 9‐tile nanoarrays could realize real‐time responses to miRNAs in complex intracellular environments owing to their characteristics of high safety, excellent physiological stability, and efficient cytoplasmic delivery properties. Hence, our strategy provides a potentially new avenue for intracellular delivery strategies and offers high‐potential orientation to apply DNA tile self‐assembly technology in basic biological research and clinical transformation.

## 4 Experimental Section

### Statistical Analysis

All experimental data were recorded from at least three independently repeated samples and denoted as mean ± standard deviation (SD). Data analysis was performed using Origin Pro 2021 software (version 9.8.0). Statistical comparisons between two samples were performed using a two‐sample *t*‐test. A significant statistical difference between two groups was considered when *p* < 0.05. The *p*‐values corresponded to ****: *p* < 0.0001, ***: *p* < 0.001, **: *p* < 0.01, *: *p* < 0.05, and ns: *p* > 0.05. Other methods are described in the Supporting Information.

## Conflict of Interest

The authors declare no conflict of interest.

## Supporting information

Supporting Information

## Data Availability

The data that support the findings of this study are available from the corresponding author upon reasonable request.
