# Supplementary material for: Size‐Controlled DNA Tile Self‐Assembly Nanostructures Through Caveolae‐Mediated Endocytosis for Signal‐Amplified Imaging of MicroRNAs in Living Cells
Source: Adv Sci (Weinh). 2023 May 15;10(21):2300614. doi: 10.1002/advs.202300614 (PMC10375201; doi:10.1002/advs.202300614)
Supplement: Supplementary file 1 — Supporting Information [file ADVS-10-2300614-s001.pdf]

ADVANCED SCIENCE
Open Access

Supporting Information

for *Adv. Sci.*, DOI 10.1002/advs.202300614

Size-Controlled DNA Tile Self-Assembly Nanostructures Through Caveolae-Mediated Endocytosis for Signal-Amplified Imaging of MicroRNAs in Living Cells

*Yanan Peng, Zhijun Gao, Bin Qiao, Dongxia Li, Huajie Pang, Xiangde Lai, Qiumei Pu, Rui Zhang, Xuan Zhao, Guangyuan Zhao, Dan Xu, Yuanyuan Wang, Yuxiang Ji, Hua Pei and Qiang Wu**

Supporting Information

Size-controlled DNA tile self-assembly nanostructures through caveolae-mediated endocytosis for signal-amplified imaging of microRNAs in living cells

*Yanan Peng, Zhijun Gao, Bin Qiao, Dongxia Li, Huajie Pang, Xiangde Lai, Qiumei Pu, Rui Zhang, Xuan Zhao, Guangyuan Zhao, Dan Xu, Yuanyuan Wang, Yuxiang Ji, Hua Pei, and Qiang Wu**

Y. Peng, Z. Gao, B. Qiao, D. Li, H. Pang, X. Lai, Q. Pu, R. Zhang, X. Zhao, G. Zhao, Y. Wang, Y. Ji, H. Pei, and Q. Wu
The Second Affiliated Hospital
School of Tropical Medicine
Hainan Medical University
Haikou 571199, P. R. China
E-mail: wuqiang001001@aliyun.com

B. Qiao, Y. Wang, and Q. Wu
Key Laboratory of Emergency and Trauma of Ministry of Education
Research Unit of Island Emergency Medicine, Chinese Academy of Medical Sciences (No. 2019RU013)
Hainan Medical University
Haikou 571199, P. R. China

D. Xu
Key Laboratory of Tropical Translational Medicine of Ministry of Education
School of Pharmacy
Hainan Medical University
Haikou 571199, P. R. China

1. Experimental Section

1.1. Materials

Oligonucleotides were synthesized from Sangon (Shanghai, CN). RNase-free ddH2O, 4 × Tris-HCl, Acryl/Bis 30% Solution (19:1), DNA Marker, Cell Counting Kit-8, and human recombinant insulin were purchased from Sangon (Shanghai, CN). 3-aminopropyl triethoxysilane (APTES), TEMED, 4% (w/w) paraformaldehyde, and Triton X-100 were purchased from Beyotime (Shanghai, CN). The 50 × TAE buffer, Hoechst 33342, Improved Minimum Essential Medium (IMEM), Dulbecco's Modified Eagle Medium (DMEM) (+ D-Glucose, + Sodium Pyruvate), FBS, PBS buffer, 0.25% Trypsin-EDTA, and Lyso-tracker Blue were purchased from ThermoFisher Scientific (Massachusetts, USA). Kaighn's Modification of Ham's F-12 Medium (F-12K) was purchased from ATCC (Virginia, USA). Magnesium chloride solution and 10% APS Solution were purchased from Aladdin (Beijing, CN). Super Red Dye was purchased from Biosharp (Anhui, CN). Nystatin and methyl-β-cyclodextrin were purchased from Med Chem Express (New Jersey, USA). F-actin Staining Kit, Cave-1 Rabbit Monoclonal Antibody, and Donkey Anti-Rabbit IgG Antibody (Alexa Fluor 568) were purchased from Abcam (Cambridge, UK). Human cervical cancer cell line Hela, human breast cancer cell line MCF-7, human lung cancer cell line A549, human liver cancer cell line HepG2, and human renal epithelial cell line 293T/17 were purchased from Procell (Wuhan, CN).

1.2. Assembly of 9-Tile Nano-Arrays

The tiles 1-9, using related ratio of 4×10^{-6} M DNA single strands in 1 × TAE/Mg^{2+} buffer, were assembled using the procedure that 95 °C for 5 min and annealing to room temperature (20 ± 5 °C) naturally. The 1 × TAE/Mg^{2+} buffer consisted of 4×10^{-4} M Tris base, 2×10^{-4} M acetic acid, 2×10^{-3} M EDTA, and 1.25×10^{-4} M magnesium acetate; pH 8.0. The 9-tile nano-arrays were assembled at the equal ratio of tiles 1-9 by annealing them from 33 °C to room temperature.

1.3. Electrophoretic Analysis

The 6% and 12% native PAGE were conducted using vertical electrophoresis systems (Bio-Rad, USA), and 1% AGE was conducted using horizontal electrophoresis systems (Tanon, China). The 1 × TAE buffer/Mg^{2+} buffer was the running buffer, and gels were run at 4 °C for 1-2 h at a constant pressure of 90-110 V. The gels were stained with Super Red Dye for 40 min, following the image via the Gel imaging analysis system (Tanon, China).

1.4. Atomic Force Microscopy

DNA samples (4 × 10^{-6} M, 10 μL) were diluted 20 times and dropped onto a freshly cleaved mica surface with positive charges after APTES treatment. Atomic Force Microscopy (AFM) images were obtained using Cyper ES (Asylum Research) with a fluid probe (BL-1C40TS, Olympus), after adding 100 μL 1 × TAE/Mg^{2+} buffer and incubating for 5 min at room temperature. The AFM images were analyzed by ImageJ 1.53 software (Czech Metrology Institute, Brno, Czech Republic).

1.5. Fluorescence Detection of MicroRNAs

The 9-tile nano-arrays (4 × 10^{-6} M, 90 μL), including 9-tile-21 or 9-tile-31, were mixed with various concentrations of miRNA-21 or miRNA-31 (10 μL), and the volume of the entire reaction system was 100 μL. After keeping the reaction system at 37 °C for 1.5 h, the fluorescence signal was collected and recorded. In the specificity validation experiment, 9-tile nano-arrays (4 × 10^{-6} M, 90 μL) were mixed with various miRNA family with a concentration of 2 × 10^{-8} M for 1.5 h at 37 °C. The fluorescence intensity was recorded at 490 nm for Alexa Fluor 488, and at 584 nm for Texas Red using Multimode Reader (SynergyHTX, BioTek, USA).

1.6. Co-Localization of 9-Tile Nano-Arrays with Caveolar Trafficking Proteins

The HeLa cells were seeded on CLSM dishes at a density of 4 × 10^4 cells/dish and cultured in the IMEM Medium with 10% FBS for 24 h at 37 °C. The cells were incubated with 9-tile-488 (2.5 × 10^{-7} M) at 37 °C for 3 h. Then, the cells were washed three times with PBS and fixed with 4% (w/w) paraformaldehyde for 20 min. The cells were permeabilized in 0.2% Triton X-

100 for 10 min and blocked in 10% FBS for 30 min. For the co-localization of 9-tile-488 with F-actin, the cells were incubated with the 1 × Red Fluorescent Phalloidin Conjugate Solution (Texas Red) for 50 min at room temperature. For the co-localization of 9-tile-488 with Cave-1, the cells were first incubated with the primary antibodies (Rabbit monoclonal, 2.28 μg mL^{-1}) for overnight at 4 °C, then incubated with the secondary antibodies (Donkey anti-Rabbit IgG, Alexa Fluor 568, 4 μg mL^{-1}) for 50 min at room temperature. The co-localization was proceeded by CLSM (FV3000, Olympus, Japan).

1.7. Analysis of the Caveolae-Mediated Cellular Uptake

The HeLa cells were seeded on 12-well plates at a density of 2×10^5 cells/well and cultured for 48 h at 37 °C, respectively. The HeLa cells were pretreated with caveolae inhibitors, including nystatin (1×10^{-5} M) or M-β-CD (2.5×10^{-3} M), for 1 h at 37 °C. Then, the cells were incubated with 9-tile-488 (2.5×10^{-7} M) at 37 °C for 3 h. Cells were collected using trypsinization after washing three times with PBS, then evaluated the fluorescence intensity via flow cytometry (FACS Melody, BD, USA).

1.8. Co-Localization with Lysosome Dye

The HeLa cells were seeded on CLSM dishes at a density of 4×10^4 cells/dish and cultured for 24 h at 37 °C. The cells were incubated with Lyso-tracker Blue (5×10^{-8} M) and 9-tile-488 (2.5×10^{-7} M) or H2-488 (2.5×10^{-7} M) for 0.5, 1, and 1.5 h at 37 °C. The cells were incubated with Lyso-tracker Blue (5×10^{-8} M) and 1-tile-488 (2.5×10^{-7} M) or 4-tile-488 (2.5×10^{-7} M) for 0.5, 1, 1.5 and 2 h at 37 °C. The co-localization was proceeded using CLSM (FV3000, Olympus, Japan).

1.9. Optimization of Imaging Conditions for 9-Tile Nano-Arrays

The HeLa cells were seeded on CLSM dishes at a density of 4×10^4 cells/dish and cultured for 24 h at 37 °C. For optimization of 9-tile nano-arrays concentration, the cells were added with 9-tile-31 in five concentration gradients (0.1, 0.2, 0.5, 1, and 2.5×10^{-7} M) for 4 h at 37 °C,

while with 9-tile-31 (1×10^{-7} M) for 1, 2, 3, and 4 h, respectively for optimization of incubation time. The MCF-7 cells were seeded on CLSM dishes at a density of 4×10^4 cells/dish and cultured in the IMEM medium with 10% FBS and human recombinant insulin (0.01 mg ml^{-1}) for 24 h at 37 °C. The cells were incubated with 9-tile-21 (1×10^{-7} M) for 1, 2, 3, and 4 h, respectively, for time optimization. The miRNAs imaging was proceeded using CLSM (FV3000, Olympus, Japan), and the fluorescence intensity was analyzed from CLSM images via ImageJ software 1.53.

1.10. Serum Stability Assay

The 9-tile nano-arrays (4×10^{-6} M) and DNA hairpins H1 (4×10^{-6} M) were incubated at 37 °C with 10% FBS for 0, 2, 4, 6, and 8 h, respectively. The products were analyzed using 6% native PAGE simultaneously.

1.11. Comparison of Imaging Effects between 9-Tile Nano-Arrays and Classical CHA

The HeLa cells were seeded on CLSM dishes at a density of 4×10^4 cells/dish and cultured for 24 h at 37 °C. The cells were incubated with 9-tile-21 (1×10^{-7} M) and 9-tile-31 (1×10^{-7} M) for 3 h at 37 °C, or with corresponding concentration of CHA-21 (hairpins H1 and H2 targeting miRNA-21) and CHA-31 (hairpins H1 and H2 targeting miRNA-31) for 3 h. Then, the resulting cells were washed twice with PBS and stained with 2.5 μg mL^{-1} Hoechst 33342 for 10 min. The miRNAs imaging was proceeded via CLSM (FV3000, Olympus, Japan).

1.12. MicroRNAs Imaging of 9-Tile Nano-Arrays in Tumor Cells

Four cell lines (A549, HepG2, MCF-7, and 293T/17 cells) were seeded on CLSM dishes at a density of 4×10^4 cells/dish and cultured for 24 h at 37 °C. The A549 cells were cultured in the Kaighn's Modification of Ham's F-12 Medium (F-12K) with 10% FBS, the HepG2 cells were cultured in the IMEM Medium with 10% FBS, and the 293T/17 cells were cultured in the Dulbecco's Modified Eagle Medium (DMEM) (+ D-Glucose, + Sodium Pyruvate) with 10% FBS. For the imaging in different cell, the cells were incubated with 9-tile-21 (1×10^{-7} M) and

9-tile-31 (1×10^{-7} M) for 3 h at 37 °C. For the imaging in identical cells with different expression levels, the cells were incubated with 9-tile-21 (1×10^{-7} M) for 3 h in miRNA-21 mimic-pretreated, in unpretreated, and in anti-miRNA-21 pretreated A549 cells. Then, the resulting cells were stained with 2.5 μg mL^{-1} Hoechst 33342 for 10 min. The miRNAs imaging was proceeded by CLSM (FV3000, Olympus, Japan).

1.13. Evaluation of Cell Viability

The HeLa cells were seeded at 5×10^{3} cells/well in 96-well plates for 24 h. Then, the cells were incubated with 9-tile nano-arrays at the condition of different concentrations (0.2, 0.5, 1, and 2.5×10^{-7} M) for 4 h, or with 9-tile nano-arrays (1×10^{-7} M) at the condition of diverse incubation times (12, 24, and 36 h). The CCK-8 solution (10 μL) was added into the cells for 4 h at 37 °C. The absorbance at 450 nm was determined with Multimode Reader (SynergyHTX, BioTek, USA).

1.14. Continuous Culture of Cancer Cells after Intracellular MicroRNAs Imaging

The MCF-7 cells or HeLa cells were collected and seeded in clean 24-well plates after intracellular miRNAs imaging. The cells were replaced with the new medium every two days and passaged every four days. The cell morphology was recorded via LM (CKX53, Olympus, Japan).

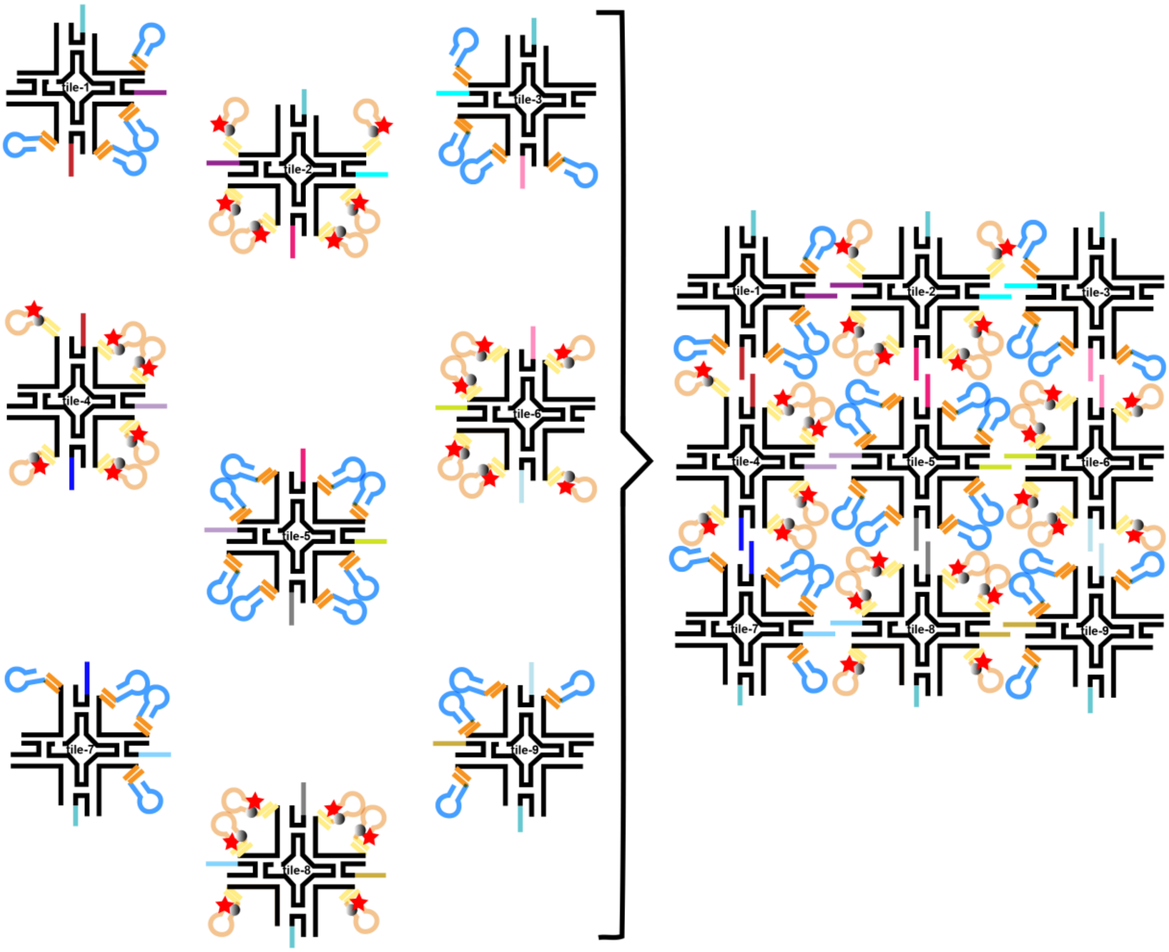

Figure S1. The structural diagram of 9-tile-21. There were nine 4PS tiles programmed with CHA hairpins to assemble into 9-tile-21. The successful assembly of 9-tile-21 attributed to the specific recognition of sticky ends. For example, tile-1 possessed four arms, including upper, lower, left and right sides. The sticky end of its right arm could only specifically recognize the left arm of tile-2, while the lower arm could only assemble with the upper arm of tile-4. Tiles 1, 3, 5, 7, and 9 were equipped with H1, while tiles 2, 4, 6, and 8 were with H2. When 9-tile assembled successfully, 24 pairs of CHA hairpins (H1 and H2) could locate in adjacent positions. Paired sticky ends indicated by the same color. Blue and orange represent hairpins H1 and H2, respectively.

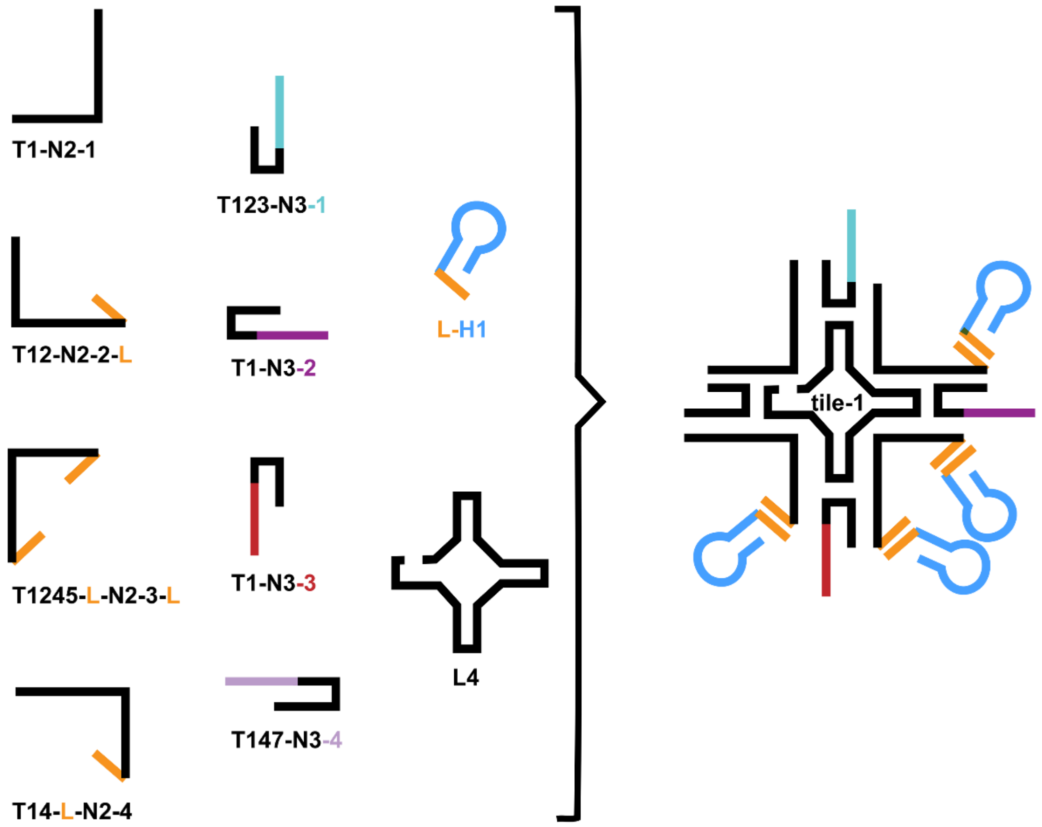

Figure S2. The structural composition of tile-1. There were ten different single-stranded DNA to assemble into tile-1. Four N2 chains, including T1-N2-1, T12-N2-2-L, T1245-L-N2-3-L, and T14-L-N2-4, formed the backbone with L4. Four N3 chains reinforced the tile-1 structure furtherly, including T123-N3-1, T1-N3-2, T1-N3-3, and T147-N3-4, where the exposed sticky ends were also the interface sequences to assemble into arrays with the other tiles. Then four L-H1 were precisely positioned to tile-1 by specific complementary pairing with linkers of N2.

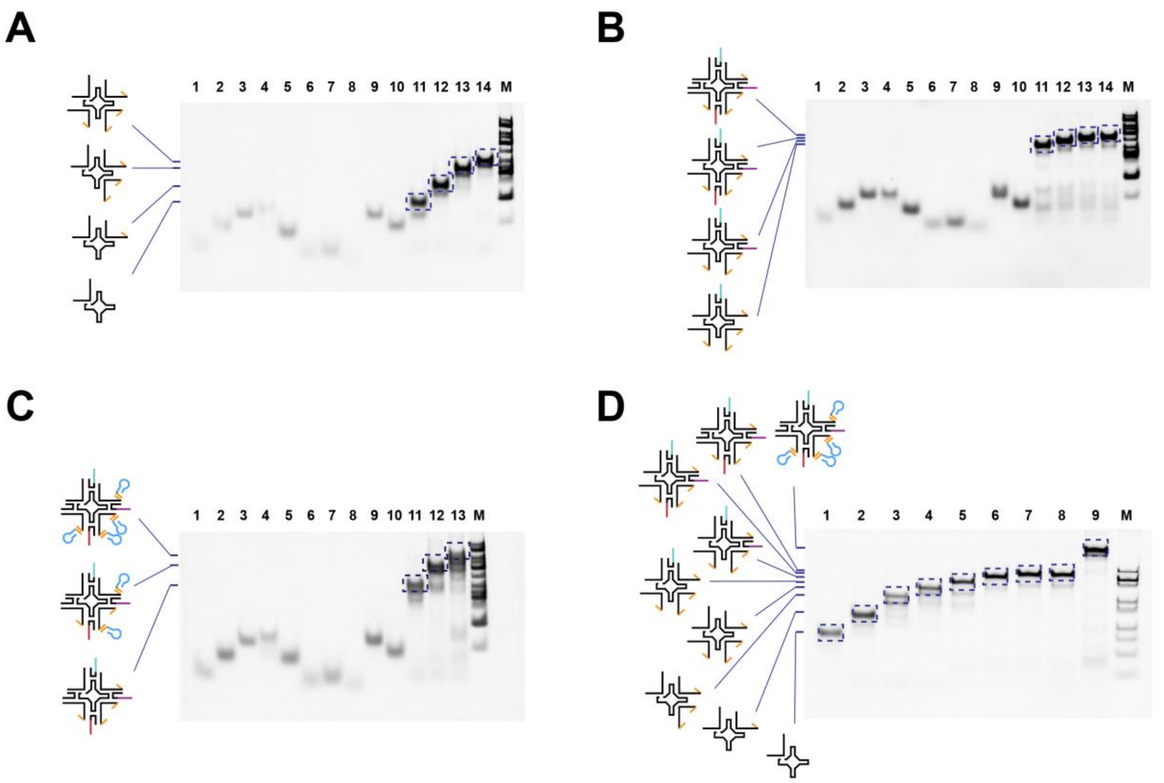

Figure S3. The 6% native PAGE analysis of tile-1. (A) Lanes 1-10, the ten DNA single strands made up of tile-1, were successively T1-N2-1, T12-N2-2-L, T1245-L-N2-3-L, T14-L-N2-4, T123-N3-1, T1-N3-2, T1-N3-3, T147-N3-4, L4, and L-H1. Lanes 11-14 were the reactants that four N2 chains added one by one in the presence of L4. (B) Lanes 1-10 were the same as (A). Lanes 11-14 were the reactants that added four N3 chains one by one based on the backbone composed of N2 and L4. (C) Lanes 1-10 were the same as (A). Lanes 11-13 were the reactants that added different ratios of L-H1 based on the backbone composed of N2, N3, and L4. (D) Lanes 1-9 were the reactants that added four N2, four N3, and four L-H1 chains one by one in the presence of L4. The DNA marker was 50-1031 bp in size (50, 100, 150, 200, 250, 300, 400, 500, 600, 700, 800, 900, 1031 bp) in figures (A), (B), and (C). The DNA marker was 25-500 bp in size (25, 50, 75, 100, 150, 200, 300, 400, 500 bp) in figure (D). The structural scheme, sample compositions, and band identities are indicated at the sides of the gel images, respectively. The concentrations of DNA substrates were constant (4 μM).

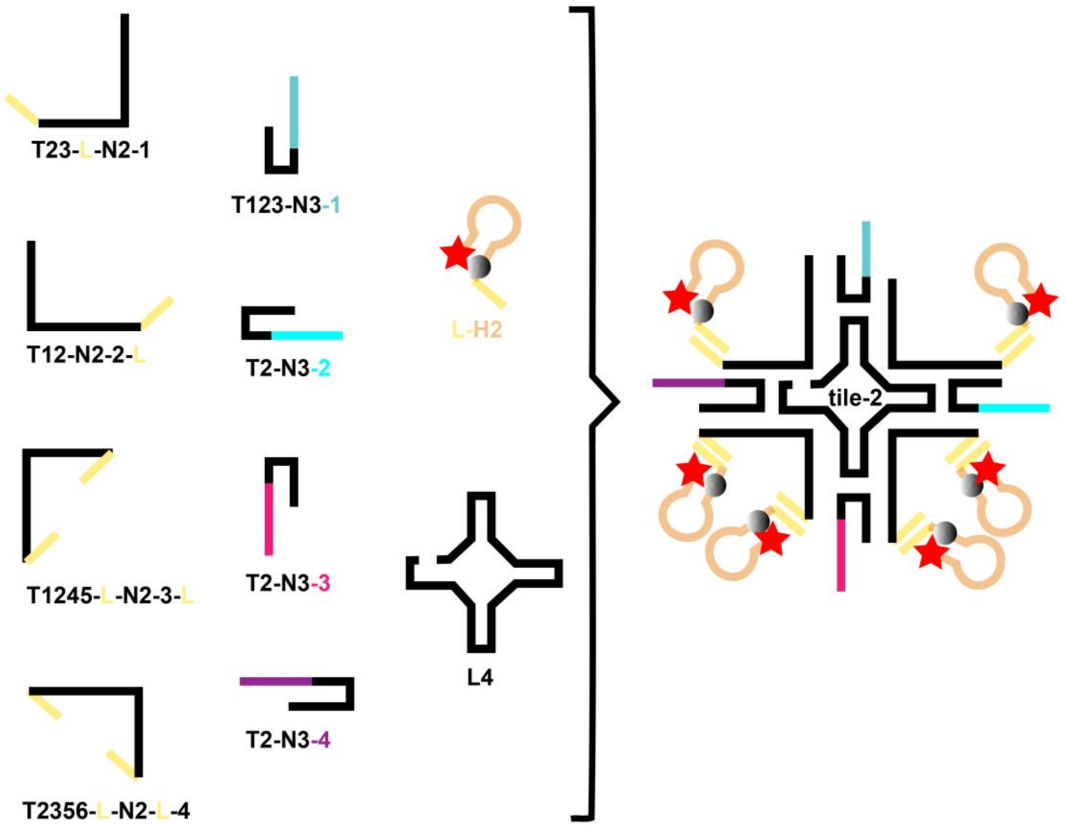

Figure S4. The structural composition of tile-2. There were ten different single-stranded DNA to assemble into tile-2. Four N2 chains, including T23-L-N2-1, T12-N2-2-L, T1245-L-N2-3-L, and T2356-L-N2-4-L, formed the backbone with L4. Four N3 chains reinforced the tile-2 structure furtherly, including T123-N3-1, T2-N3-2, T2-N3-3, and T2-N3-4, where the exposed sticky ends were also the interface sequences to assemble into arrays with the other tiles. Then six L-H2 were precisely positioned to tile-2 by specific complementary pairing with linkers of N2.

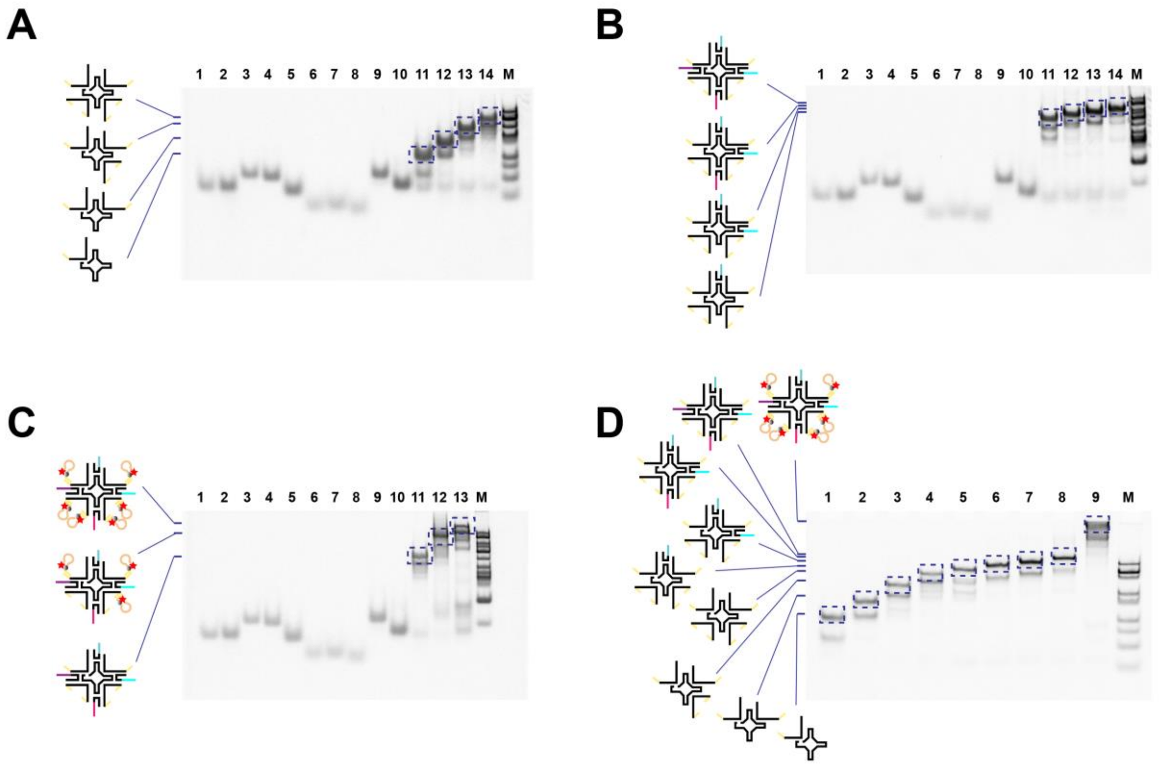

Figure S5. The 6% native PAGE analysis of tile-2. (A) Lanes 1-10, the ten DNA single strands made up of tile-2, were successively T23-L-N2-1, T12-N2-2-L, T1245-L-N2-3-L, T2356-L-N2-4-L, T123-N3-1, T2-N3-2, T2-N3-3, T2-N3-4, L4, and L-H2. Lanes 11-14 were the reactants that four N2 chains added one by one in the presence of L4. (B) Lanes 1-10 were the same as (A). Lanes 11-14 were the reactants that added four N3 chains one by one based on the backbone composed of N2 and L4. (C) Lanes 1-10 were the same as (A). Lanes 11-13 were the reactants that added different ratios of L-H2 based on the backbone composed of N2, N3, and L4. (D) Lanes 1-9 were the reactants that added four N2, four N3, and six L-H2 chains one by one in the presence of L4. The DNA marker was 50-1031 bp in size (50, 100, 150, 200, 250, 300, 400, 500, 600, 700, 800, 900, 1031 bp) in figures (A), (B), and (C). The DNA marker was 25-500 bp in size (25, 50, 75, 100, 150, 200, 300, 400, 500 bp) in figure (D). The structural scheme, sample compositions, and band identities are indicated at the sides of the gel images, respectively. The concentrations of DNA substrates were constant (4 μM).

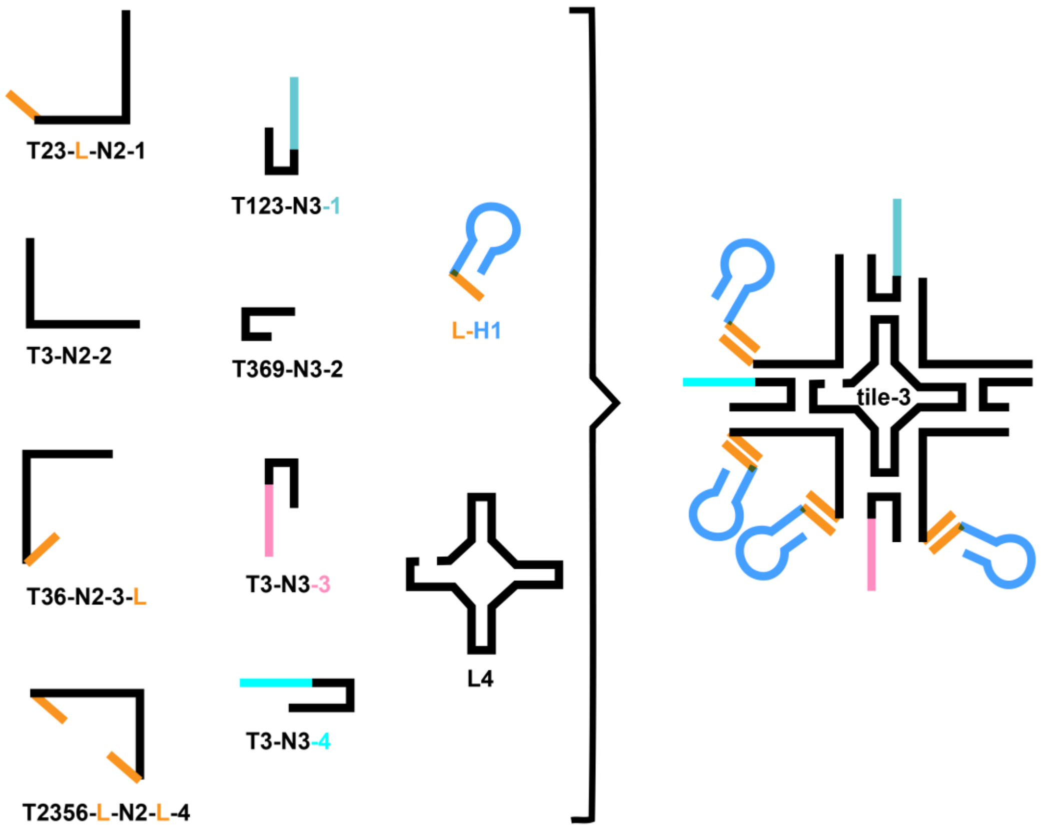

Figure S6. The structural composition of tile-3. There were ten different single-stranded DNA to assemble into tile-3. Four N2 chains, including T23-L-N2-1, T3-N2-2, T36-N2-3-L, and T2356-L-N2-4-L, formed the backbone with L4. Four N3 chains reinforced the tile-3 structure furtherly, including T123-N3-1, T369-N3-2, T3-N3-3, and T3-N3-4, where the exposed sticky ends were also the interface sequences to assemble into arrays with the other tiles. Then four L-H1 were precisely positioned to tile-3 by specific complementary pairing with linkers of N2.

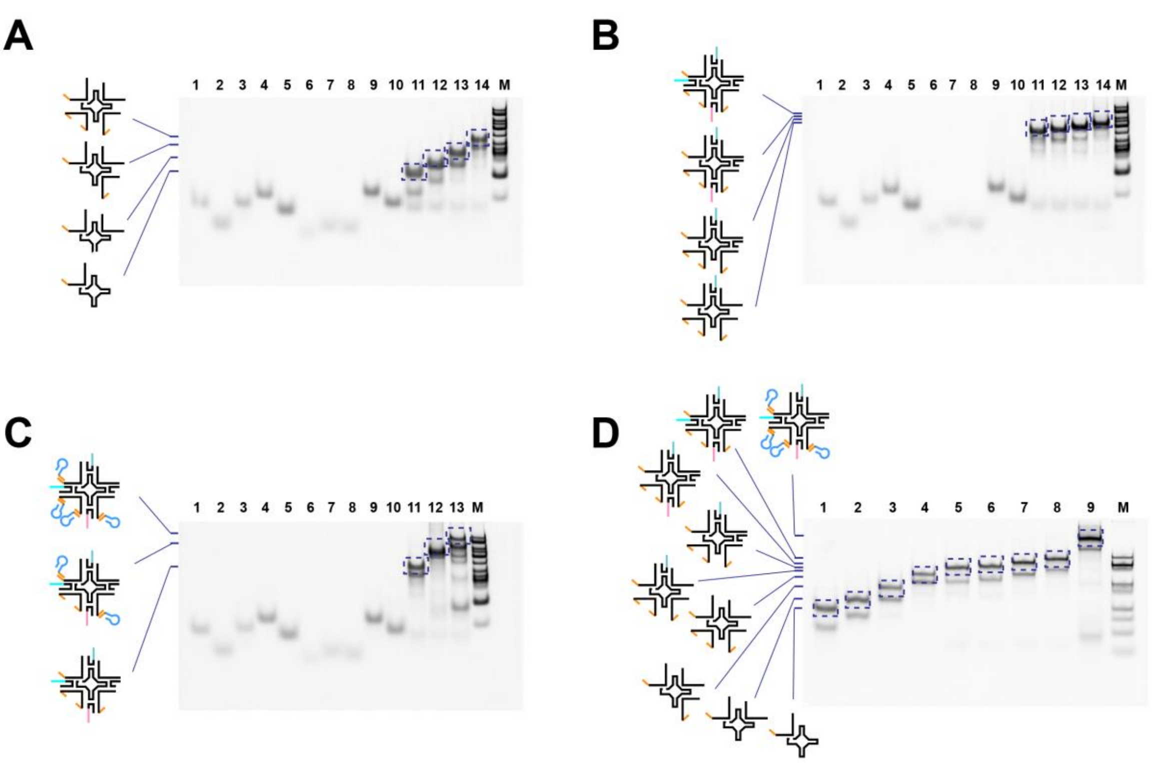

Figure S7. The 6% native PAGE analysis of tile-3. (A) Lanes 1-10, the ten DNA single strands made up of tile-3, were successively T23-L-N2-1, T3-N2-2, T36-N2-3-L, T2356-L-N2-4-L, T123-N3-1, T369-N3-2, T3-N3-3, T3-N3-4, L4, and L-H1. Lanes 11-14 were the reactants that four N2 chains added one by one in the presence of L4. (B) Lanes 1-10 were the same as (A). Lanes 11-14 were the reactants that four N3 chains added one by one based on the backbone of N2 and L4. (C) Lanes 1-10 were the same as (A). Lanes 11-13 were the reactants that added different ratios of L-H1 based on the backbone composed of N2, N3, and L4. (D) Lanes 1-9 were the reactants that four N2, four N3, and four L-H1 chains added one by one in the presence of L4. The DNA marker was 50-1031 bp in size (50, 100, 150, 200, 250, 300, 400, 500, 600, 700, 800, 900, 1031 bp) in figures (A), (B), and (C). The DNA marker was 25-500 bp in size (25, 50, 75, 100, 150, 200, 300, 400, 500 bp) in figure (D). The structural scheme, sample compositions, and band identities are indicated at the sides of the gel images, respectively. The concentrations of DNA substrates were constant (4 µM).

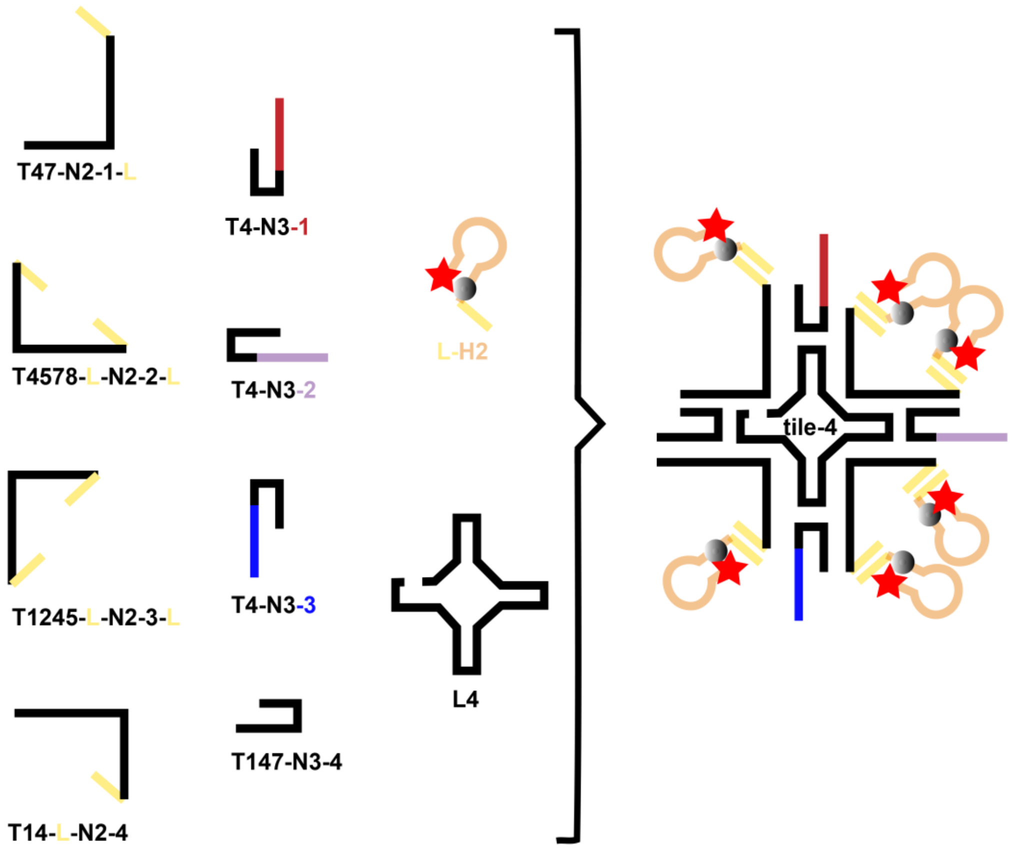

Figure S8. The structural composition of tile-4. There were ten different single-stranded DNA to assemble into tile-4. Four N2 chains, including T47-N2-1-L, T4578-L-N2-2-L, T1245-L-N2-3-L, and T14-L-N2-4, formed the backbone with L4. Four N3 chains reinforced the tile-4 structure furtherly, including T4-N3-1, T4-N3-2, T4-N3-3, and T147-N3-4, where the exposed sticky ends were also the interface sequences to assemble into arrays with the other tiles. Then six L-H2 were precisely positioned to tile-4 by specific complementary pairing with linkers of N2.

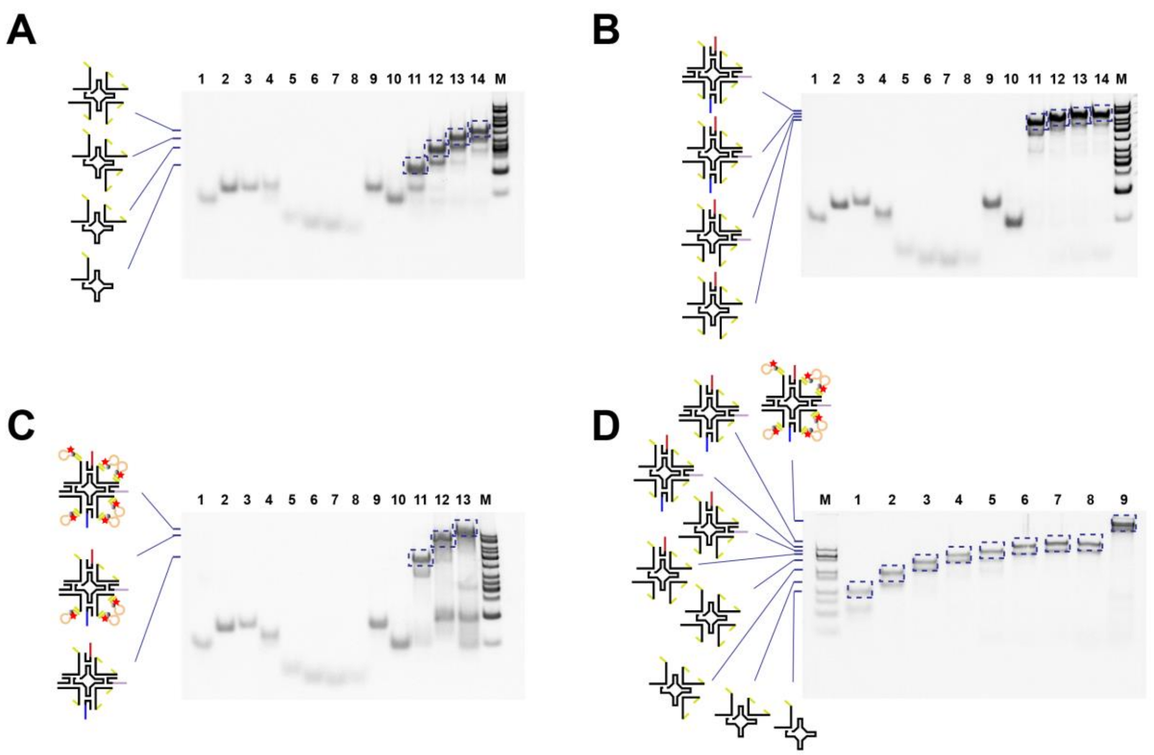

Figure S9. The 6% native PAGE analysis of tile-4. (A) Lanes 1-10, the ten DNA single strands made up of tile-4, were successively T47-N2-1-L, T4578-L-N2-2-L, T1245-L-N2-3-L, T14-L-N2-4, T4-N3-1, T4-N3-2, T4-N3-3, T147-N3-4, L4, and L-H2. Lanes 11-14 were the reactants that four N2 chains added one by one in the presence of L4. (B) Lanes 1-10 were the same as (A). Lanes 11-14 were the reactants that four N3 chains added one by one based on the backbone of N2 and L4. (C) Lanes 1-10 were the same as (A). Lanes 11-13 were the reactants that added different ratios of L-H2 based on the backbone composed of N2, N3, and L4. (D) Lanes 1-9 were the reactants that four N2, four N3, and six L-H2 chains added one by one in the presence of L4. The DNA marker was 50-1031 bp in size (50, 100, 150, 200, 250, 300, 400, 500, 600, 700, 800, 900, 1031 bp) in figures (A), (B), and (C). The DNA marker was 25-500 bp in size (25, 50, 75, 100, 150, 200, 300, 400, 500 bp) in figure (D). The structural scheme, sample compositions, and band identities are indicated at the sides of the gel images, respectively. The concentrations of DNA substrates were constant (4 μM).

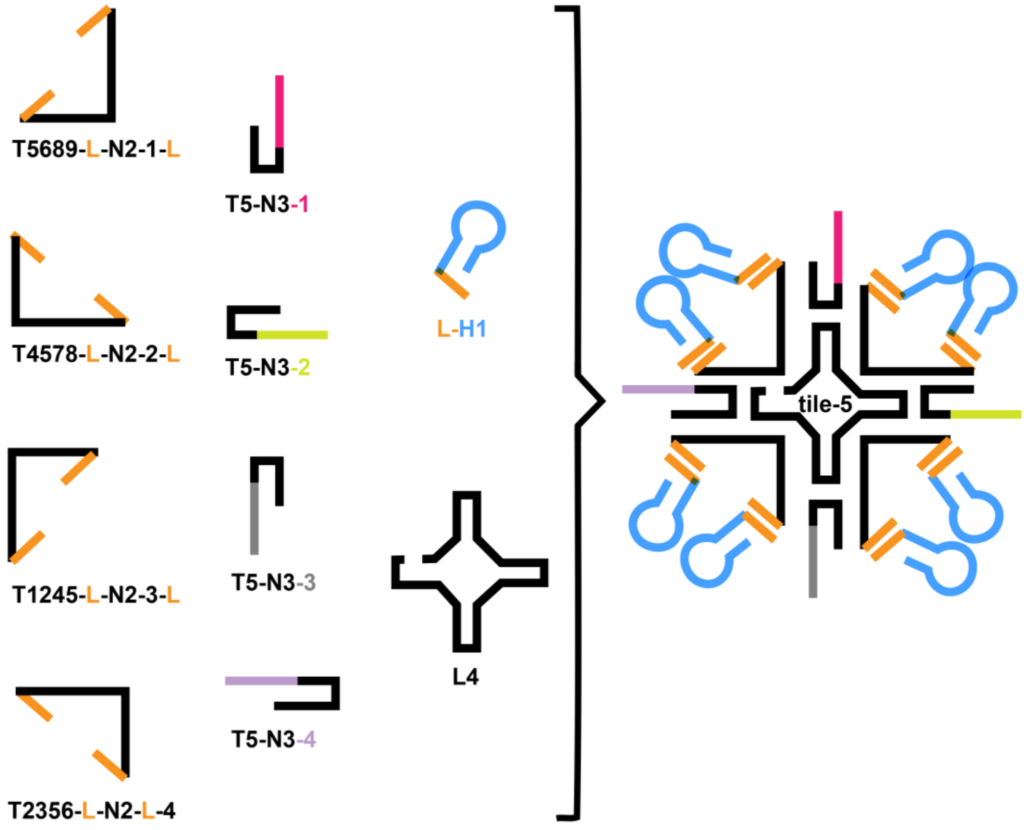

Figure S10. The structural composition of tile-5. There were ten different single-stranded DNA to assemble into tile-5. Four N2 chains, including T5689-L-N2-1-L, T4578-L-N2-2-L, T1245-L-N2-3-L, and T2356-L-N2-4-L, formed the backbone with L4. Four N3 chains reinforced the tile-5 structure furtherly, including T5-N3-1, T5-N3-2, T5-N3-3, and T5-N3-4, where the exposed sticky ends were also the interface sequences to assemble into arrays with the other tiles. Then eight L-H1 were precisely positioned to tile-5 by specific complementary pairing with linkers of N2.

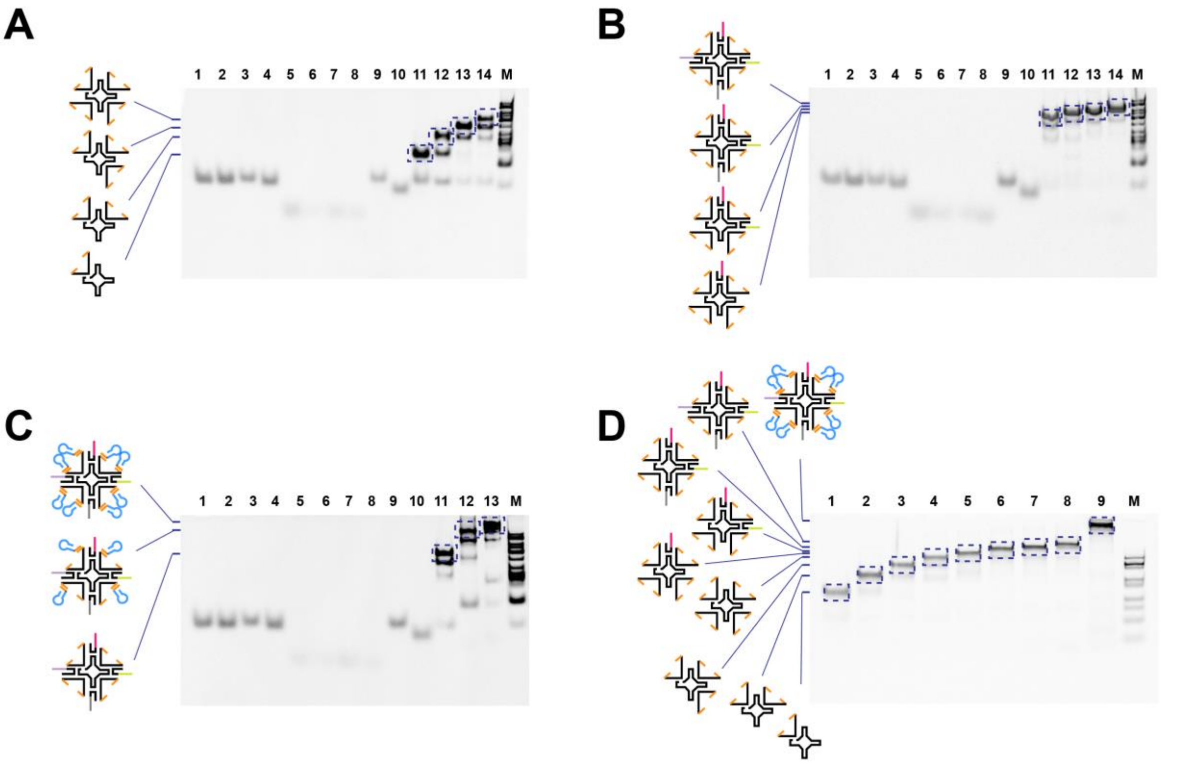

Figure S11. The 6% native PAGE analysis of tile-5. (A) Lanes 1-10, the ten DNA single strands made up of tile-5, were successively T5689-L-N2-1-L, T4578-L-N2-2-L, T1245-L-N2-3-L, T2356-L-N2-4-L, T5-N3-1, T5-N3-2, T5-N3-3, T5-N3-4, L4, and L-H1. Lanes 11-14 were the reactants that four N2 chains added one by one in the presence of L4. (B) Lanes 1-10 were the same as (A). Lanes 11-14 were the reactants that four N3 chains added one by one based on the backbone of N2 and L4. (C) Lanes 1-10 were the same as (A). Lanes 11-13 were the reactants that added different ratios of L-H1 based on the backbone composed of N2, N3, and L4. (D) Lanes 1-9 were the reactants that four N2, four N3, and eight L-H1 chains added one by one in the presence of L4. The DNA marker was 50-1031 bp in size (50, 100, 150, 200, 250, 300, 400, 500, 600, 700, 800, 900, 1031 bp) in figures (A), (B), and (C). The DNA marker was 25-500 bp in size (25, 50, 75, 100, 150, 200, 300, 400, 500 bp) in figure (D). The structural scheme, sample compositions, and band identities are indicated at the sides of the gel images, respectively. The concentrations of DNA substrates were constant (4 μM).

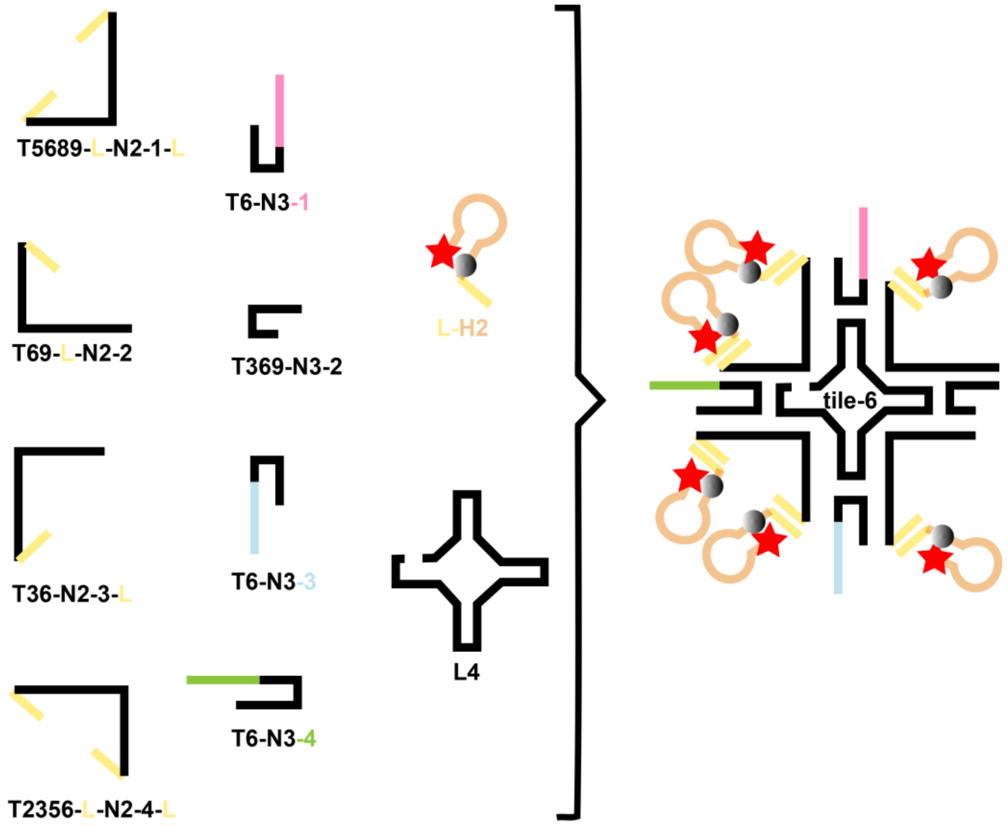

Figure S12. The structural composition of tile-6. There were ten different single-stranded DNA to assemble into tile-6. Four N2 chains, including T5689-L-N2-1-L, T69-L-N2-2, T36-N2-3-L, and T2356-L-N2-4-L, formed the backbone with L4. Four N3 chains reinforced the tile-6 structure furtherly, including T6-N3-1, T369-N3-2, T6-N3-3, and T6-N3-4, where the exposed sticky ends were also the interface sequences to assemble into arrays with the other tiles. Then six L-H2 were precisely positioned to tile-5 by specific complementary pairing with linkers of N2.

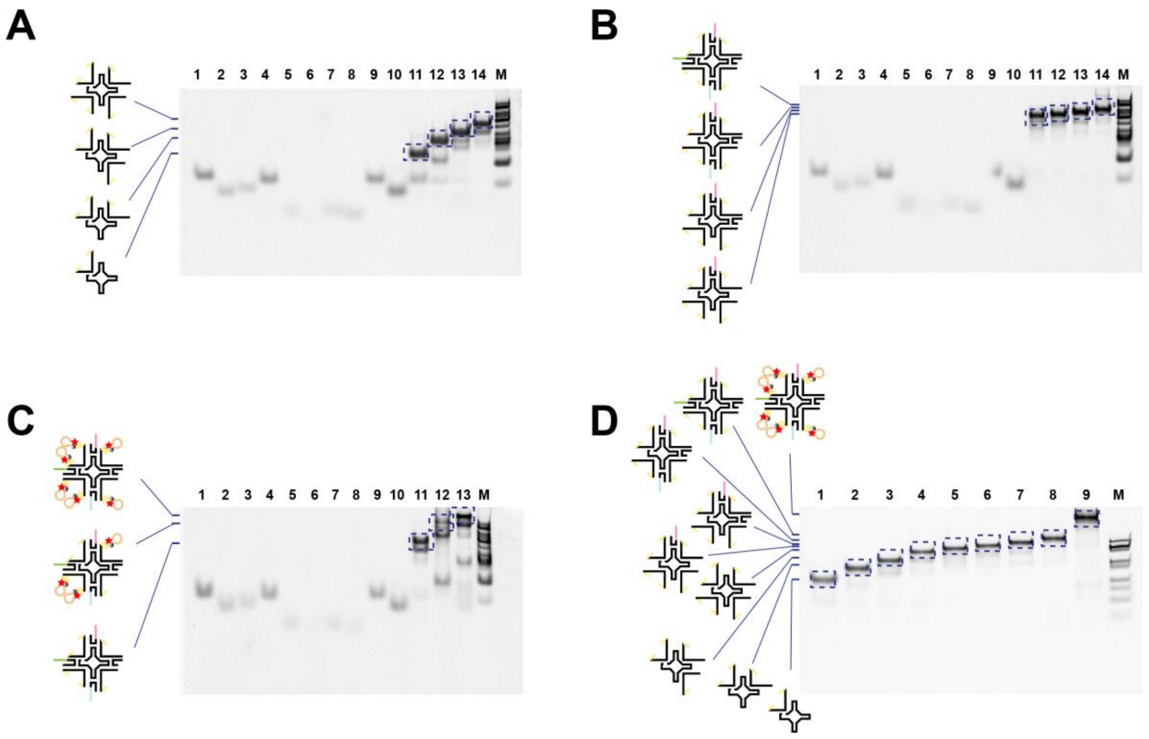

Figure S13. The 6% native PAGE analysis of tile-6. (A) Lanes 1-10, the ten DNA single strands made up of tile-6, were successively T5689-L-N2-1-L, T69-L-N2-2, T36-N2-3-L, T2356-L-N2-4-L, T6-N3-1, T369-N3-2, T6-N3-3, T6-N3-4, L4, and L-H2. Lanes 11-14 were the reactants that four N2 chains added one by one in the presence of L4. (B) Lanes 1-10 were the same as (A). Lanes 11-14 were the reactants that four N3 chains added one by one based on the backbone of N2 and L4. (C) Lanes 1-10 were the same as (A). Lanes 11-13 were the reactants that added different ratios of L-H2 based on the backbone composed of N2, N3, and L4. (D) Lanes 1-9 were the reactants that four N2, four N3, and six L-H2 chains added one by one in the presence of L4. The DNA marker was 50-1031 bp in size (50, 100, 150, 200, 250, 300, 400, 500, 600, 700, 800, 900, 1031 bp) in figures (A), (B), and (C). The DNA marker was 25-500 bp in size (25, 50, 75, 100, 150, 200, 300, 400, 500 bp) in figure (D). The structural scheme, sample compositions, and band identities are indicated at the sides of the gel images, respectively. The concentrations of DNA substrates were constant (4 μM).

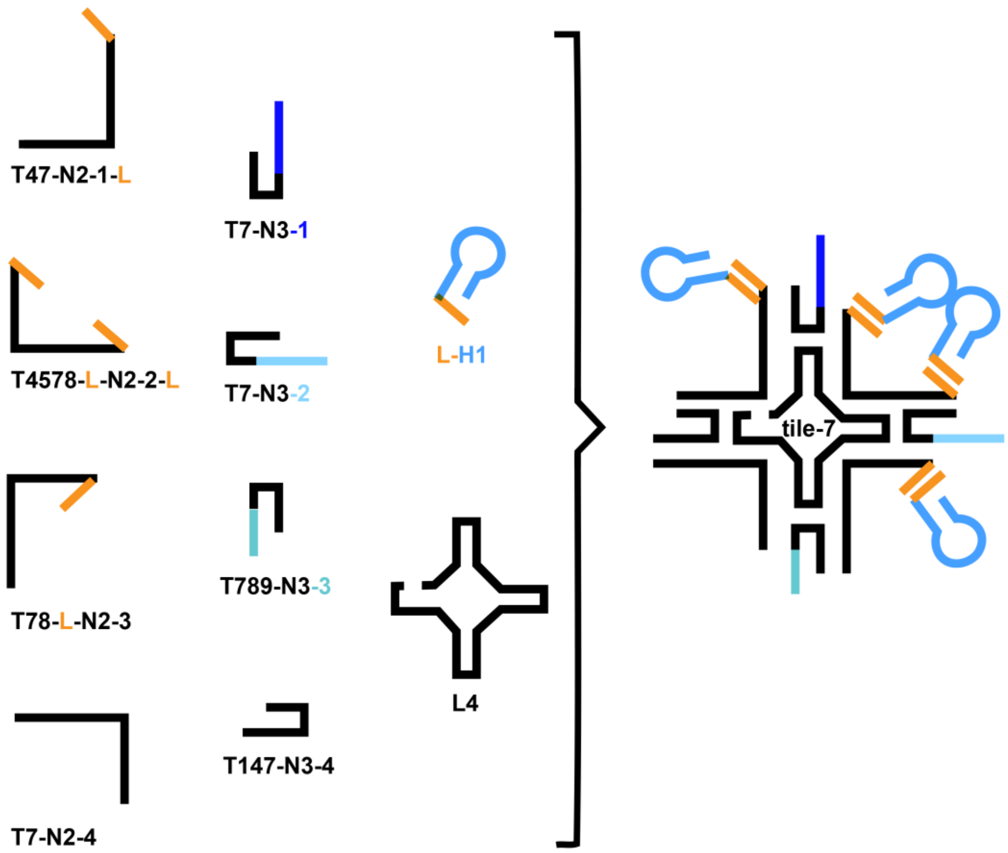

Figure S14. The structural composition of tile-7. There were ten different single-stranded DNA to assemble into tile-7. Four N2 chains, including T47-N2-1-L, T4578-L-N2-2-L, T78-L-N2-3, and T7-N2-4, formed the backbone with L4. Four N3 chains reinforced the tile-7 structure furtherly, including T7-N3-1, T7-N3-2, T789-N3-3, and T147-N3-4, where the exposed sticky ends were also the interface sequences to assemble into arrays with the other tiles. Then four L-H1 were precisely positioned to tile-7 by specific complementary pairing with linkers of N2.

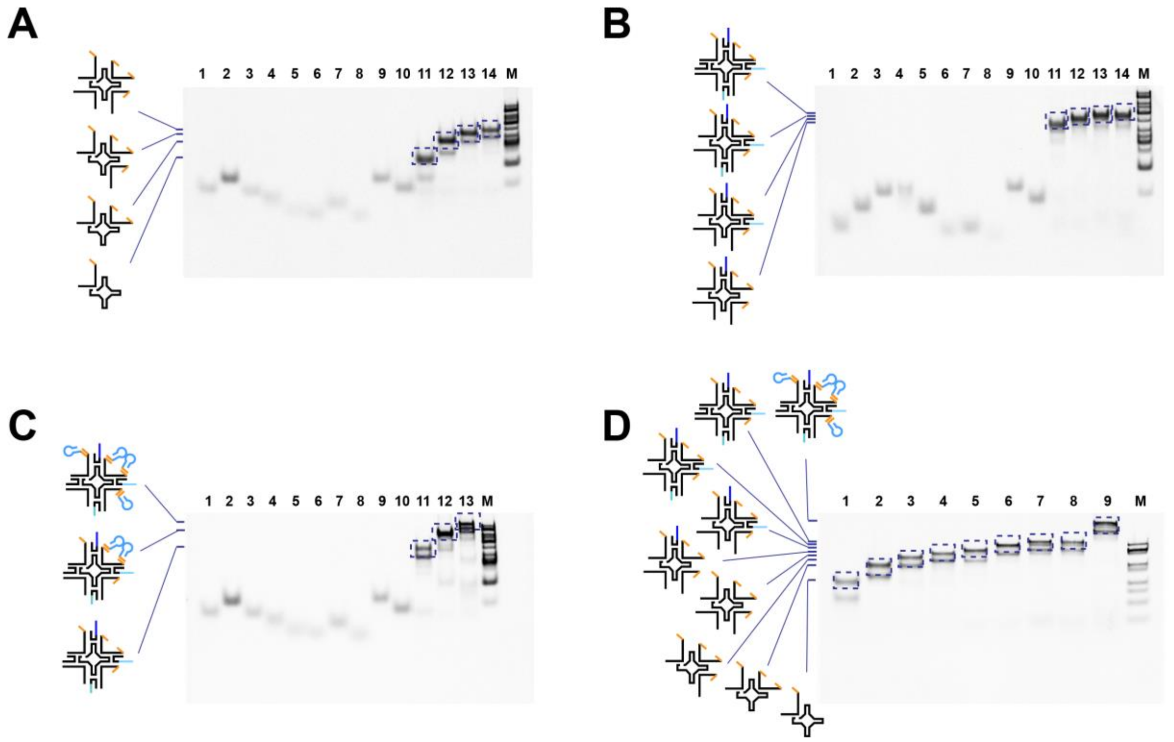

Figure S15. The 6% native PAGE analysis of tile-7. (A) Lanes 1-10, the ten DNA single strands made up of tile-7, were successively T47-N2-1-L, T4578-L-N2-2-L, T78-L-N2-3, T7- N2-4, T7-N3-1, T7-N3-2, T789-N3-3, T147-N3-4, L4, and L-H1. Lanes 11-14 were the reactants that four N2 chains added one by one in the presence of L4. (B) Lanes 1-10 were the same as (A). Lanes 11-14 were the reactants that four N3 chains added one by one based on the backbone of N2 and L4. (C) Lanes 1-10 were the same as (A). Lanes 11-13 were the reactants that added different ratios of L-H1 based on the backbone composed of N2, N3, and L4. (D) Lanes 1-9 were the reactants that four N2, four N3, and four L-H1 chains added one by one in the presence of L4. The DNA marker was 50-1031 bp in size (50, 100, 150, 200, 250, 300, 400, 500, 600, 700, 800, 900, 1031 bp) in figures (A), (B), and (C). The DNA marker was 25-500 bp in size (25, 50, 75, 100, 150, 200, 300, 400, 500 bp) in figure (D). The structural scheme, sample compositions, and band identities are indicated at the sides of the gel images, respectively. The concentrations of DNA substrates were constant (4 μM).

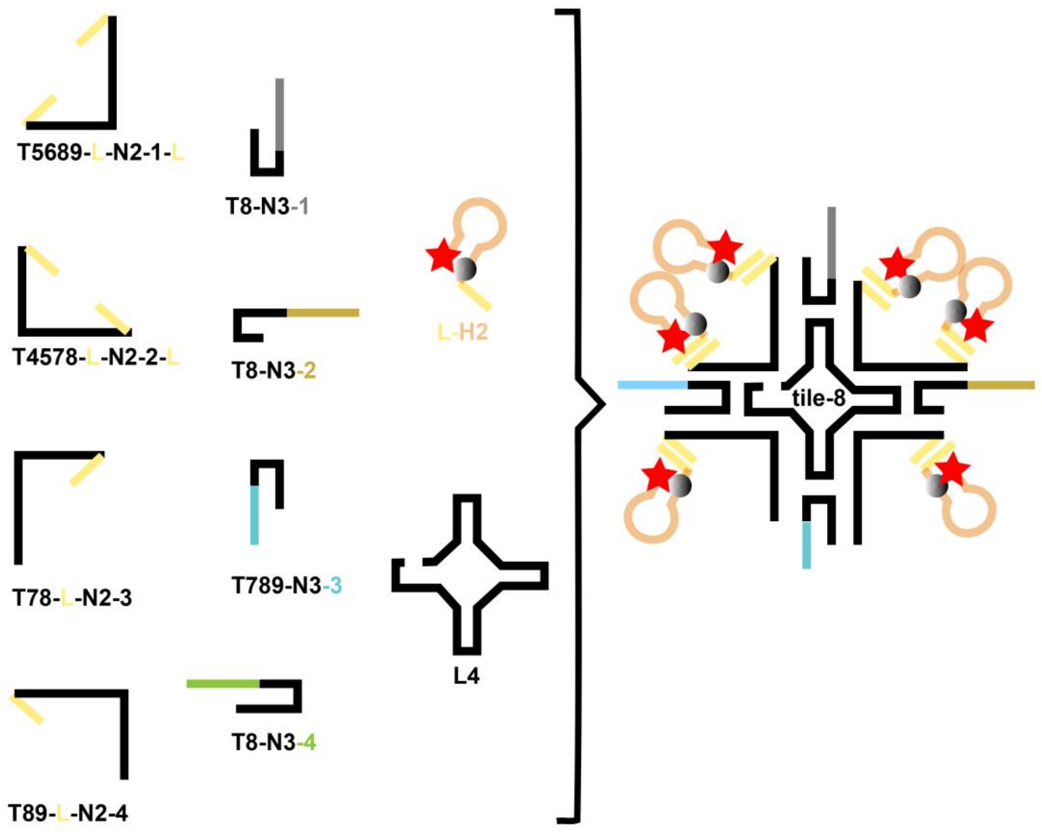

Figure S16. The structural composition of tile-8. There were ten different single-stranded DNA to assemble into tile-8. Four N2 chains, including T5689-l-N2-1-L, T4578-L-N2-2-L, T78-L-N2-3, and T89-L-N2-4, formed the backbone with L4. Four N3 chains reinforced the tile-8 structure furtherly, including T8-N3-1, T8-N3-2, T789-N3-3, and T8-N3-4, where the exposed sticky ends were also the interface sequences to assemble into arrays with the other tiles. Then six L-H2 were precisely positioned to tile-8 by specific complementary pairing with linkers of N2.

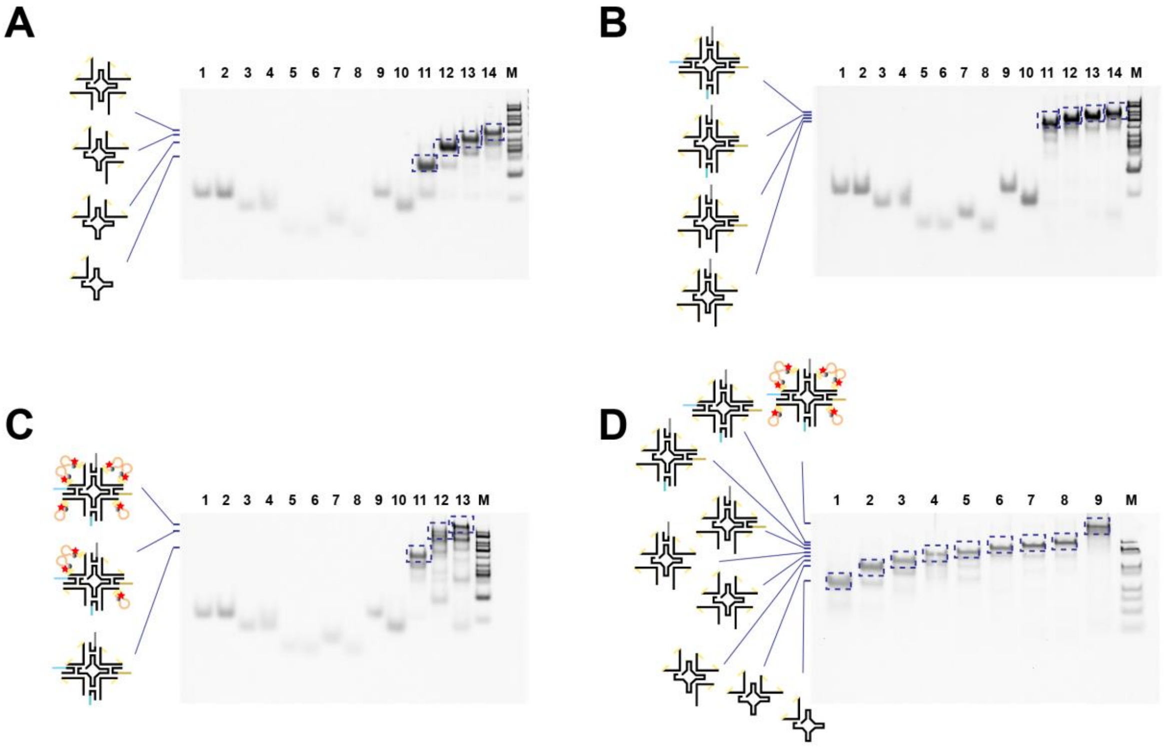

Figure S17. The 6% native PAGE analysis of tile-8. (A) Lanes 1-10, the ten DNA single strands made up of tile-8, were successively T5689-l-N2-1-L, T4578-L-N2-2-L, T78-L-N2-3, T89-L-N2-4, T8-N3-1, T8-N3-2, T789-N3-3, T8-N3-4, L4, and L-H2. Lanes 11-14 were the reactants that four N2 chains added one by one in the presence of L4. (B) Lanes 1-10 were the same as (A). Lanes 11-14 were the reactants that four N3 chains added one by one based on the backbone of N2 and L4. (C) Lanes 1-10 were the same as (A). Lanes 11-13 were the reactants that added different ratios of L-H2 based on the backbone composed of N2, N3, and L4. (D) Lanes 1-9 were the reactants that four N2, four N3, and six L-H2 chains added one by one in the presence of L4. The DNA marker was 50-1031 bp in size (50, 100, 150, 200, 250, 300, 400, 500, 600, 700, 800, 900, 1031 bp) in figures (A), (B), and (C). The DNA marker was 25-500 bp in size (25, 50, 75, 100, 150, 200, 300, 400, 500 bp) in figure (D). The structural scheme, sample compositions, and band identities are indicated at the sides of the gel images, respectively. The concentrations of DNA substrates were constant (4 μM).

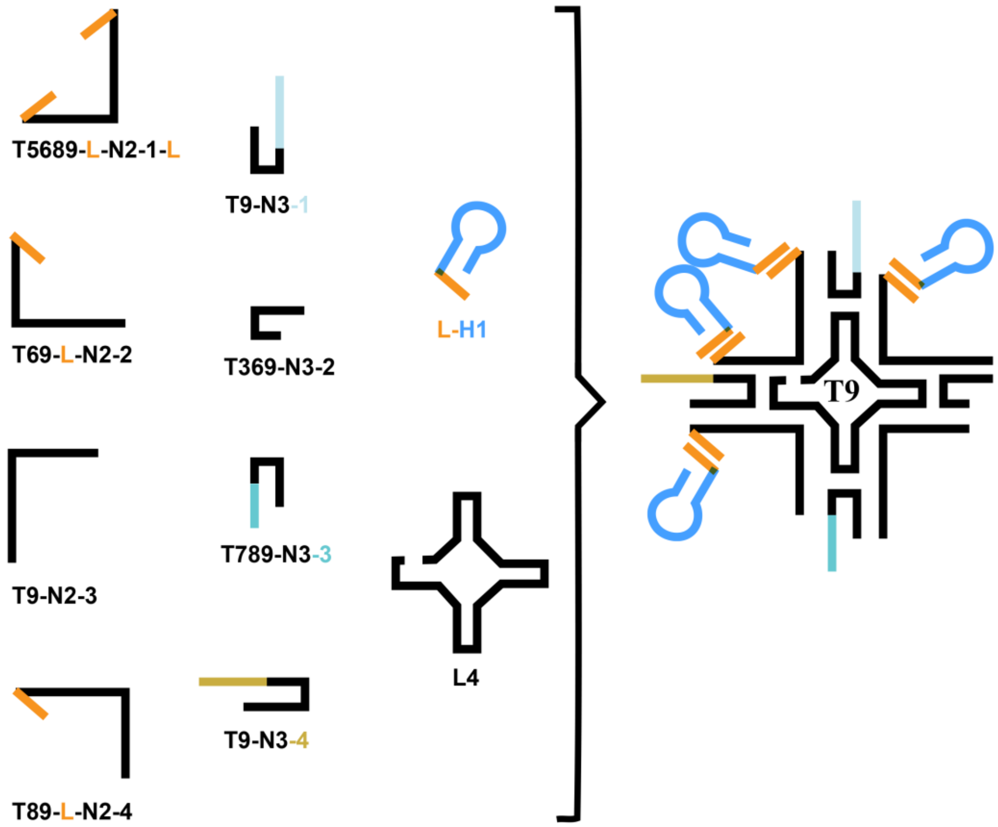

Figure S18. The structural composition of tile-9. There were ten different single-stranded DNA to assemble into tile-9. Four N2 chains, including T5689-l-N2-1-L, T69-L-N2-2, T9-N2-3, and T89-L-N2-4, formed the backbone with L4. Four N3 chains reinforced the tile-9 structure furtherly, including T9-N3-1, T369-N3-2, T789-N3-3, and T9-N3-4, where the exposed sticky ends were also the interface sequences to assemble into arrays with the other tiles. Then four L-H1 were precisely positioned to tile-9 by specific complementary pairing with linkers of N2.

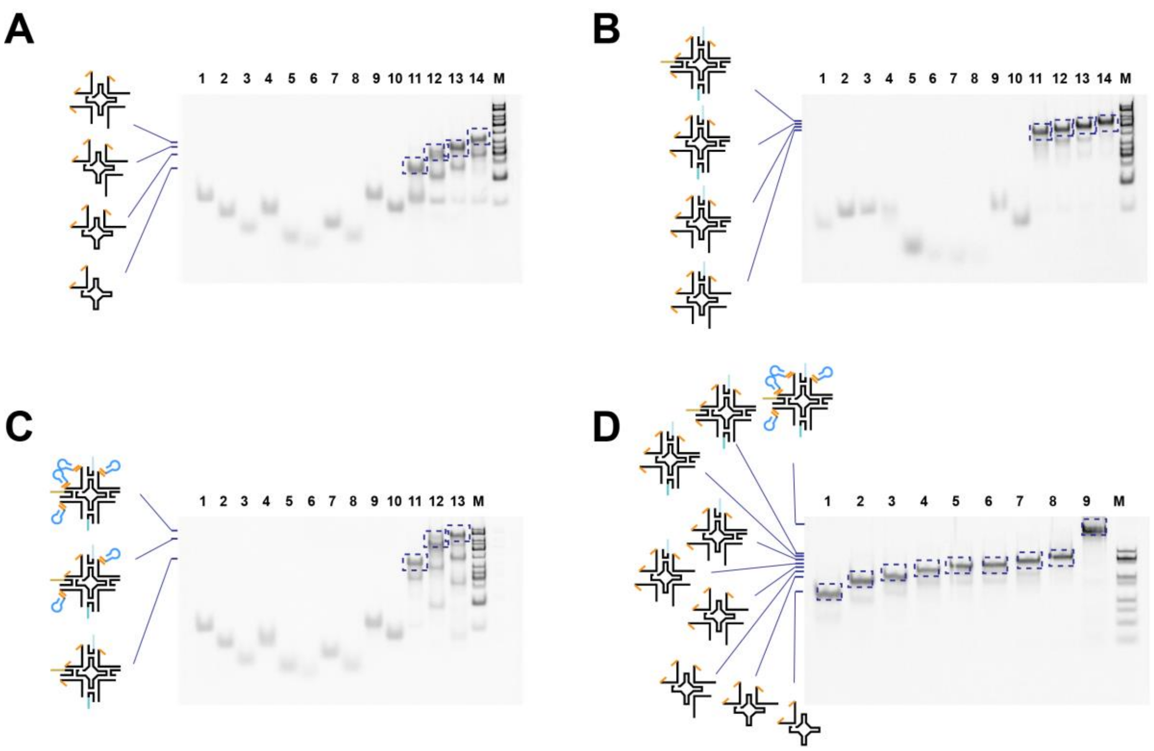

Figure S19. The 6% native PAGE analysis of tile-9. (A) Lanes 1-10, the ten DNA single strands made up of tile-9, were successively T5689-l-N2-1-L, T69-L-N2-2, T9 -N2-3, T89-L-N2-4, T9-N3-1, T369-N3-2, T789-N3-3, T9-N3-4, L4, and L-H1. Lanes 11-14 were the reactants that four N2 chains added one by one in the presence of L4. (B) Lanes 1-10 were the same as (A). Lanes 11-14 were the reactants that four N3 chains added one by one based on the backbone of N2 and L4. (C) Lanes 1-10 were the same as (A). Lanes 11-13 were the reactants that added different ratios of L-H1 based on the backbone composed of N2, N3, and L4. (D) Lanes 1-9 were the reactants that four N2, four N3, and four L-H1 chains added one by one in the presence of L4. The DNA marker was 50-1031 bp in size (50, 100, 150, 200, 250, 300, 400, 500, 600, 700, 800, 900, 1031 bp) in figures (A), (B), and (C). The DNA marker was 25-500 bp in size (25, 50, 75, 100, 150, 200, 300, 400, 500 bp) in figure (D). The structural scheme, sample compositions, and band identities are indicated at the sides of the gel images, respectively. The concentrations of DNA substrates were constant (4 μM).

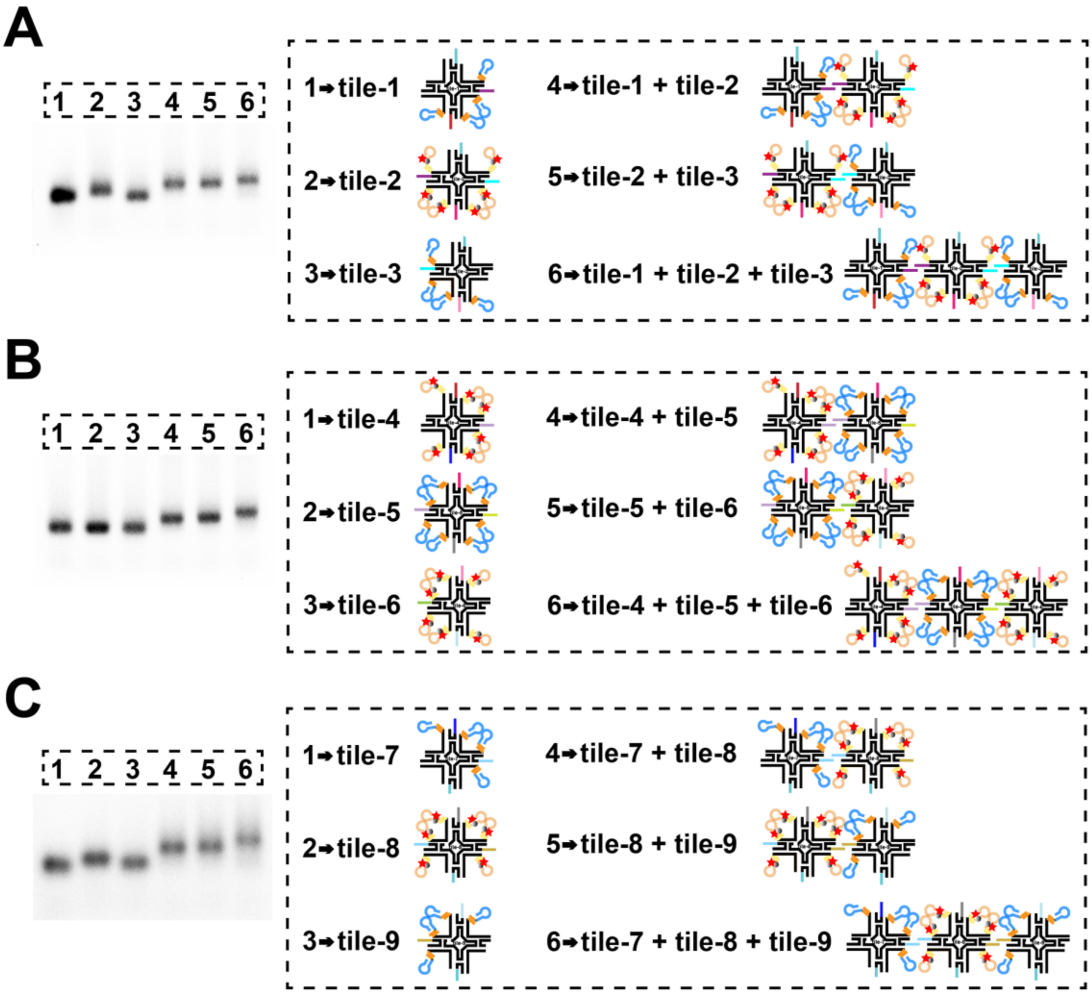

Figure S20. The 1% AGE analysis of the horizontal assembly process for 9-tile. (A) The assembly process among tile-1, tile-2, and tile-3. Lanes 1 was tile-1, lane 2 was tile-2, lane 3 was tile-3, lane 4 was the assembly products of tile-1 and tile-2, lane 5 was the assembly products of tile-2 and tile-3, and lane 6 was the assembly products of tile-1, tile-2, and tile-3. (B) The assembly process among tile-4, tile-5, and tile-6. Lanes 1 was tile-4, lane 2 was tile-5, lane 3 was tile-6, lane 4 was the assembly products of tile-4 and tile-5, lane 5 was the assembly products of tile-5 and tile-6, and lane 6 was the assembly products of tile-4, tile-5, and tile-6. (C) The assembly process among tile-7, tile-8, and tile-9. Lanes 1 was tile-7, lane 2 was tile-8, lane 3 was tile-9, lane 4 was the assembly products of tile-7 and tile-8, lane 5 was the assembly products of tile-8 and tile-9, and lane 6 was the assembly products of tile-7, tile-8, and tile-9. The structural scheme, sample compositions, and band identities are indicated at the sides of the gel images, respectively. The concentrations of DNA substrates were constant (4 μM).

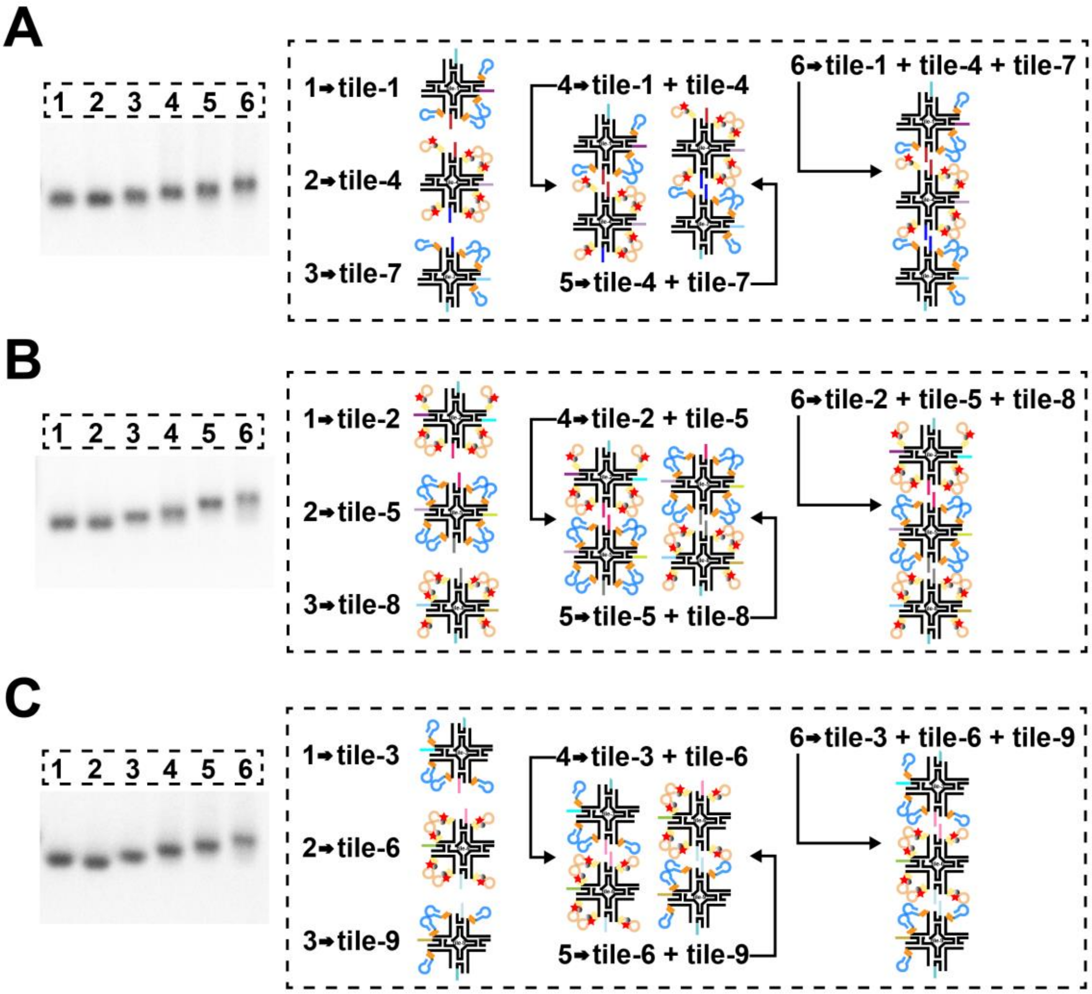

Figure S21. The 1% AGE analysis of the vertical assembly process for 9-tile. (A) The assembly process among tile-1, tile-4, and tile-7. Lanes 1 was tile-1, lane 2 was tile-4, lane 3 was tile-7, lane 4 was the assembly products of tile-1 and tile-4, lane 5 was the assembly products of tile-4 and tile-7, and lane 6 was the assembly products of tile-1, tile-4, and tile-7. (B) The assembly process among tile-2, tile-5, and tile-8. Lanes 1 was tile-2, lane 2 was tile-5, lane 3 was tile-8, lane 4 was the assembly products of tile-2 and tile-5, lane 5 was the assembly products of tile-5 and tile-8, and lane 6 was the assembly products of tile-2, tile-5, and tile-8. (C) The assembly process among tile-3, tile-6, and tile-9. Lanes 1 was tile-3, lane 2 was tile-6, lane 3 was tile-9, lane 4 was the assembly products of tile-3 and tile-6, lane 5 was the assembly products of tile-6 and tile-9, and lane 6 was the assembly products of tile-3, tile-6, and tile-9. The structural scheme, sample compositions, and band identities are indicated at the sides of the gel images, respectively. The concentrations of DNA substrates were constant (4 μM).

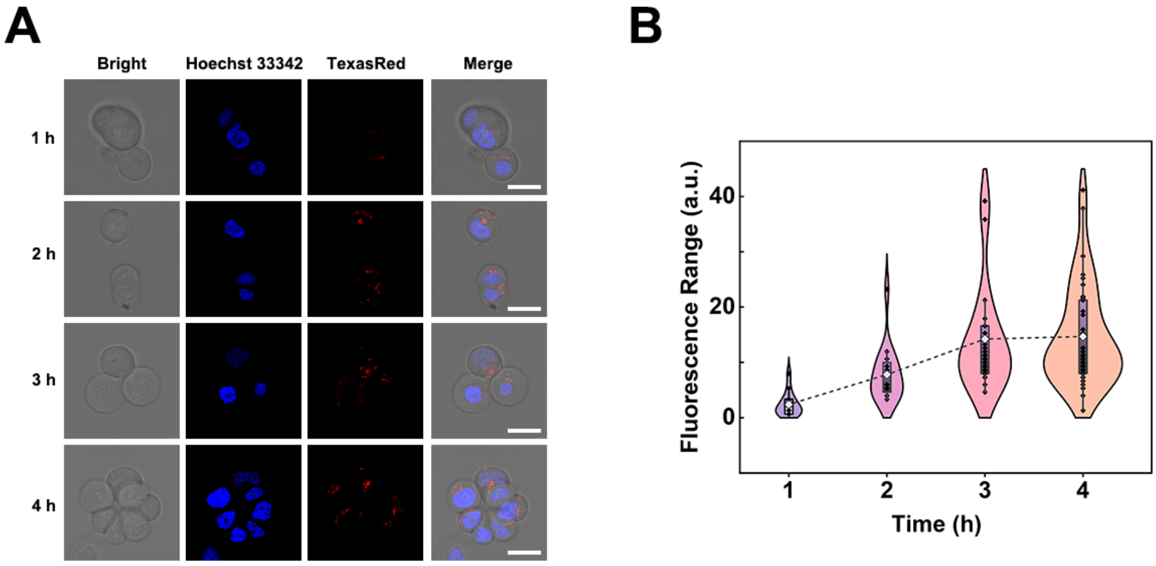

Figure S22. Optimization of incubate time for 9-tile-21 imaging in MCF-7 cells. A) Confocal laser scanning microscopy (CLSM) imaging of MCF-7 cells treated with 9-tile-21 at 1-h intervals. B) The statistical violin plot analysis of the fluorescence signal distribution of CLSM images (n = 3) for each incubate time of 9-tile-21. The dotted line was the mean values connected for each group. All scale bars correspond to 20 µm.

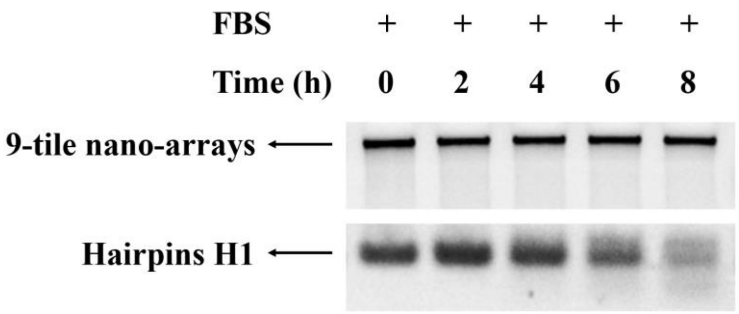

Figure S23. The 6% native PAGE analysis of 10% fetal bovine serum (FBS) degradation products for 9-tile nano-arrays and hairpins H1 within 8 h. The concentrations of DNA substrates were constant (4 μM).

Table S1. Oligonucleotide sequences involved in the research

Name	Sequence (5′-3′)
T1-N2-1	GCTACCCTGTAGACCCGTTTCTCACGGGACGCCTC
T12-N2-2-L	GAGCACCGGATCTAAGTCGTTCCGACGGACGAACCGCTGT TTTTTTTTTTTTTTTTTTTT
T1245-L-N2-3-L	TTTTTTTTTTTTTTTTTTTTGATGCCCTGACCGAGTCCCCAT AGATGGTACTTGAACCTGATTTTTTTTTTTTTTTTTTT
T14-L-N2-4	TTTTTTTTTTTTTTTTTTTCAAGTGCCTGGTCGAAATGCACA CGTAGGACATTCA
T123-N3-1	TTTGAGGCGTGGTGCTCTTT
T1-N3-2	AACAGCGGTTCGTGGGCATCATAGCACGCTC
T1-N3-3	TCAGGTTCAAGTAGGCACTTGAAACAGAAGCT
T147-N3-4	TTTTGAATGTGGGTAGCTTT
L4	ACACCTACGTGTGTTTTCATTTCGACCACCATCTATGGTTTT GGACTCGGTCACCGTCGGAACTTTTGACTTAGATCCCCCG TGAGAATTTTACGGGTC
L4-488	ACACCTACGTGTGTTTTCATTTCGACCACCATCTATGGTTTT GGACTCGGTCACCGTCGGAACTTTTGACTTAGATCCCCCG TGAGAATTTTACGGGTC-Alexa Flour 488
MiRNA-21-L-H1	AAAAAAAAAAAAAAAAAAATCAACATCAGTCTGATAAGC TACTACAACTGGCGGGGTAGCTTATCAGACT
T23-L-N2-1	TTTTTTTTTTTTTTTTTTTGCCTACCCTGTAGACCCGTTTCTC ACGGGACGCCTC
T2356-L-N2-4-L	TTTTTTTTTTTTTTTTTTTCAAGTGCCTGGTCGAAATGCACA CGTAGGACATTCATTGGCATTTTTTTTTTTTTTTTTTT
T2-N3-2	AACAGCGGTTCGTGGGCATCATGAGTAAAGGG
T2-N3-3	TCAGGTTCAAGTAGGCACTTGAAGCCGACTTA
T2-N3-4	TGCCAATGAATGTGGGTAGGCGAGCGTGCTA
MiRNA-21-L-H2	TAAGCTACCCCGCCAG/iTexRddT/TGTAGTAGCTTATCAGAC TCTACAAC/iBHQ2dT/GGCGGGGAAAAAAAAAAAAAAAAAAAA AA
T3-N2-2	GAGCACCGGATCTAAGTCGTTCCGACGGACGAACC
T36-N2-3-L	TGATGCCCTGACCGAGTCCCCATAGATGGTACTTGAACCTG

	ATTTTTTTTTTTTTTTTTTT
T369-N3-2	TTTGGTTCGTGGGCATCTTT
T3-N3-3	TCAGGTTCAAGTAGGCACTTGTTAGACCGACC
T3-N3-4	TGCCAATGAATGTGGGTAGGCCCCTTTACTCA
T47-N2-1-L	GCTACCCTGTAGACCCGTTTCTCACGGGACGCCTCGGATC<br>CTTTTTTTTTTTTTTTTTTT
T4578-L-N2-2-L	TTTTTTTTTTTTTTTTTTTCGAGCACCGGATCTAAGTCGTTC<br>CGACGGACGAACCGCTGTT TTTTTTTTTTTTTTTTTTT
T4-N3-1	GGATCCGAGGCGTGGTGCTCGAGCTTCTGTTT
T4-N3-2	AACAGCGGTTCGTGGGCATCACACCACGAGA
T4-N3-3	TCAGGTTCAAGTAGGCACTTGCTGGAACTTCG
T5689-L-N2-1-L	TTTTTTTTTTTTTTTTTTTGCCTACCCTGTAGACCCGTTTCTC<br>ACGGGACGCCTCGGATCCTTTTTTTTTTTTTTTTTTT
T5-N3-1	GGATCCGAGGCGTGGTGCTCGTAAGTCGGCTT
T5-N3-2	AACAGCGGTTCGTGGGCATCAGTGATCATAGG
T5-N3-3	TCAGGTTCAAGTAGGCACTTGAGTTGTCACAA
T5-N3-4	TGCCAATGAATGTGGGTAGGCTCTCGTGGTG
T69-L-N2-2	TTTTTTTTTTTTTTTTTTTCGAGCACCGGATCTAAGTCGTTC<br>CGACGGACGAACC
T6-N3-1	GGATCCGAGGCGTGGTGCTCGGGTCGGTCTAA
T6-N3-3	TCAGGTTCAAGTAGGCACTTGTCCGATACGCC
T6-N3-4	TGCCAATGAATGTGGGTAGGCCCTATGATCAC
T78-L-N2-3	TTTTTTTTTTTTTTTTTTTTGATGCCCTGACCGAGTCCCCAT<br>AGATGGACAAGCC
T7-N2-4	AAGTGCCTGGTCGAAATGCACACGTAGGACATTC
T7-N3-1	GGATCCGAGGCGTGGTGCTCGCGAAGTTCCAG
T7-N3-2	AACAGCGGTTCGTGGGCATCACGAGAAGGAAT
T789-N3-3	TTTGGCTTGTGGCACTTTTTTTTTGACTTAAACCCTAGGA
T89-L-N2-4	TTTTTTTTTTTTTTTTTTTAAGTGCCTGGTCGAAATGCACAC<br>GTAGGACATTCATTGGCA
T8-N3-1	GGATCCGAGGCGTGGTGCTCGTTGTGACAACT
T8-N3-2	AACAGCGGTTCGTGGGCATCAAAAGCCCTTAT
T8-N3-4	TGCCAATGAATGTGGGTAGGCATTCCTTCTCG
T9-N2-3	TGATGCCCTGACCGAGTCCCCATAGATGGACAAGCC

T9-N3-1	GGATCCGAGGCGTGGTGCTCGGGCGTATCGGA
T9-N3-4	TGCCAATGAATGTGGGTAGGCATAAGGGCTTT
MiRNA-31-L-H1	AGCTATGCCAGCATCTTGCCTGTGAACTCGGAAGGCAAGA TGCAAAAAAAAAAAAAAAAAAAA
MiRNA-31-L-H2	AAAAAAAAAAAAAAAAAAAATTGCCT/iBHQ1dT/CCGAGTTC ACAGGCAAGATGCGTGAACTCGGA-Alexa Fluor 488
MiRNA-21	UAGCUUAUCAGACUGAUGUUGA
MiRNA-31	AGGCAAGAUGCUGGCAUAGCU
MiRNA-25	AGGCGGAGACUUGGGCAAUUG
MiRNA-133	UUUGGUCCCCUUCAACCAGCUG
MiRNA-19b	AGUUUUGCAGGUUUGCAUCCAGC
Anti-miRNA-21	TCAACATCAGTCTGATAAGCTA
MiRNA-21 mimic	TAGCTTATCAGACTGATGTTGA

The fluorescence groups are highlighted in red. The quench groups are highlighted in blue.

Table S2. Comparison of microRNAs imaging performance of the strategies based on DNA nanostructure.

Name	Working concentration	Optimal time of miRNAs imaging	Reference
DNA Tetrahedral	3×10^{-7} M	5 h	[1]
CCD circuit	/	10 h	[2]
ES-AuNP	/	8 h	[3]
DNF	1×10^{-7} M	5 h	[4]
DNA nanomachine	$0.01/20/2 \times 10^{-6}$ M mix	8 h	[5]
9-tile nano-arrays	1×10^{-7} M	3 h	This study

CCD circuit: controlled catalytic DNA circuit, ES-AuNP: AuNP-mediated self-protected DNAzyme walker, DNF: DNA nanocage framework.

Table S3. Thermodynamic parameters of two-state melting hybridization between paired tiles on 9-tile nano-arrays.

Paired Tiles	ΔG_{37} (kcal mol^{-1})	ΔH (kcal mol^{-1})	ΔS (cal mol^{-1} K^{-1})	T_m (°C)
tile-1 and tile-2	-11.6	-76.1	-208.0	46.3
tile-1 and tile-4	-10.1	-77.4	-217.0	40.0
tile-2 and tile-3	-9.9	-81.8	-231.8	39.1
tile-2 and tile-5	-11.6	-79.4	-218.7	45.8
tile-3 and tile-6	-11.1	-80.3	-223.2	43.7
tile-4 and tile-5	-10.9	-74.3	-204.4	43.6
tile-4 and tile-7	-11.0	-83.1	-232.5	43.2
tile-5 and tile-6	-9.3	-79.0	-224.6	36.8
tile-5 and tile-8	-10.1	-77.9	-218.7	39.9
tile-6 and tile-9	-12.9	-83.9	-229.0	50.6
tile-7 and tile-8	-10.2	-79.4	-223.0	40.4
tile-8 and tile-9	-9.8	-74.9	-210.0	38.7

ΔG_{37} is the Gibbs free energy at 37 °C, ΔH is the enthalpy, ΔS is the entropy, and the Tm value is the melting temperature. The energy rules are as follows: DNA concentrations = 1×10^{-6} M, $[Na^{+}] = 1 \times 10^{-2}$ M, $[Mg^{2+}] = 1.25 \times 10^{-2}$ M, and the temperature is 37 °C. The simulated calculation results of thermodynamic parameters are acquired from The UNAFold Web Server.

Reference:

[1] Z. Zhou, D. Fan, J. Wang, Y. S. Sohn, R. Nechushtai, I. Willner, *Small* **2021**, *17*, 2007355.

[2] S. He, S. Yu, R. Li, Y. Chen, Q. Wang, Y. He, X. Liu, F. Wang, *Angew. Chem. Int. Ed.* **2022**, *61*, e202206529.

[3] Y. Gao, S. Zhang, C. Wu, Q. Li, Z. Shen, Y. Lu, Z.-S. Wu, *ACS Nano* **2021**, *15*, 19211-19224.

[4] X. Li, F. Yang, S. Li, R. Yuan, Y. Xiang, *Anal. Chem.* **2022**, *94*, 9927-9933.

[5] J. Y. Wang, H. D. Li, P. Q. Ma, Y. Zhou, B. C. Yin, B. C. Ye, *Biosens. and Bioelectron.* **2023**, *220*, 114828.
